# CSNK2A1-mediated MAX phosphorylation upregulates HMGB1 and IL-6 expression in cholangiocarcinoma progression

**DOI:** 10.1097/HC9.0000000000000144

**Published:** 2023-06-22

**Authors:** Bing Yang, Jing Zhang, Jiaohong Wang, Wei Fan, Lucía Barbier-Torres, Xi Yang, Monica Anne R. Justo, Ting Liu, Yongheng Chen, Justin Steggerda, Komal Ramani, Shelly C. Lu, Heping Yang

**Affiliations:** 1 Department of Medicine, Karsh Division of Gastroenterology and Hepatology, Cedars-Sinai Medical Center, Los Angeles, California, USA; 2 Department of Geriatric Endocrinology and Metabolism, Key Laboratory of Precision Medicine in Cardio-Cerebrovascular Diseases Control and Prevention and Clinical Research Center for Cardio-Cerebrovascular Diseases, The First Affiliated Hospital of Guangxi Medical University, Nanning, Guangxi, China; 3 Department of Oncology, Tongji Hospital, Tongji Medical College, Huazhong University of Science and Technology, Wuhan, China; 4 Department of General Surgery, Cedars-Sinai Medical Center, Los Angeles, California, USA; 5 Department of Gastroenterology, Key Laboratory of Cancer Proteomics of Chinese Ministry of Health, Xiangya Hospital, Central South University, Changsha, Hunan, China; 6 Department of Oncology, NHC Key Laboratory of Cancer Proteomics & Laboratory of Structural Biology, Xiangya Hospital, Central South University, Changsha, Hunan, China

## Abstract

We established a novel diethylnitrosamine (DEN) -induced mouse model that reflected the progression of cholangiocarcinoma (CCA) from atypical cystic hyperplasia.

**Methods:**

BALB/c mice were administered DEN by oral gavage. Cells isolated from livers were analyzed for expression of CSNK2A1, MAX and MAX-interacting proteins. Human CCA cell lines (MzChA-1, HuCCT1), normal human cholangiocyte (H69), human hepatic stellate cells (LX-2), macrophages (RAW 264.7), and primary hepatic cells were used for cellular and molecular biology assays.

**Results:**

Expression of MAX, CSNK2A1, C-MYC, β-catenin, HMGB1, and IL-6 was upregulated in hepatic cells from CCA liver tissue. The half-life of MAX is higher in CCA cells, and this favors their proliferation. Overexpression of MAX increased growth, migration, and invasion of MzChA-1, whereas silencing of MAX had the opposite effect. MAX positively regulated IL-6 and HMGB1 through paracrine signaling in HepG2, LX2, and RAW cells and autocrine signaling in MzChA-1 cells. CSNK2A1-mediated MAX phosphorylation shifts MAX-MAX homodimer to C-MYC-MAX and β-catenin-MAX heterodimers and increases the HMGB1 and IL-6 promoter activities. Increase of MAX phosphorylation promotes cell proliferation, migration, invasion, and cholangiocarcinogenesis. The casein kinase 2 inhibitor CX-4945 induces cell cycle arrest and inhibits cell proliferation, migration, invasion, and carcinogenesis in MzChA-1 cells through the downregulation of CSNK2A1, MAX, and MAX-interaction proteins.

**Conclusion:**

C-MYC-MAX and β-catenin-MAX binding to E-box site or β-catenin-MAX bound to TCFs/LEF1 enhanced HMGB1 or IL-6 promoter activities, respectively. IL-6 and HMGB1 secreted by hepatocytes, HSCs, and KCs exert paracrine effects on cholangiocytes to promote cell growth, migration, and invasion and lead to the progression of cholangiocarcinogenesis. CX-4945 provides perspectives on therapeutic strategies to attenuate progression from atypical cystic hyperplasia to cholangiocarcinogenesis.

## Graphical abstract

The schematic diagram shows a novel cross talk between MYC-associated factor X (MAX) and CSNK2A1, C-MYC, β-catenin, high-mobility group box-1 (HMGB1), and IL-6 in CCA. On the one hand, CSNK2A1 activates and CX-4945 inhibit MAX phosphorylation. The former leads to shift of MAX-MAX homodimer to MAX-C-MYC/MAX-β-catenin heterodimers and promotes HMGB1 transcription in MzChA-1 cell. On the other hand, high expression of CSNK2A1 and MAX in hepatocytes, KCs, and HSCs and cholangiocytes in liver tissues with CCA leads to increase in MAX phosphorylation and promotes paracrine and autocrine signaling of HMGB1 and IL-6. HMGB1 and IL-6 can regulate the occurrence of biliary proliferation, atypical hyperplasia, cholangiomas, and CCA.

**Figure:**
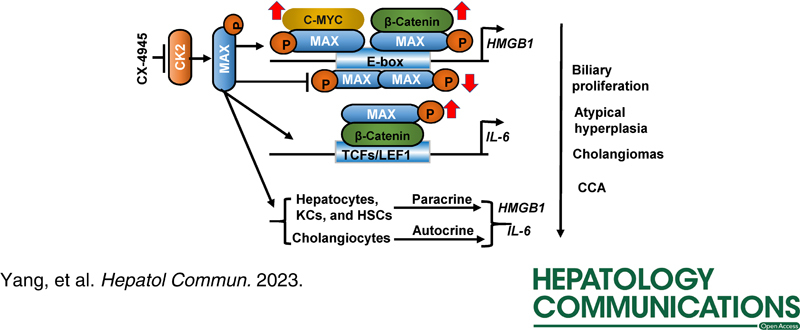

## INTRODUCTION

Cholangiocarcinoma (CCA) is a common invasive cancer of the intrahepatic and extrahepatic bile ducts. According to the anatomical location, it is divided into 3 subtypes: intrahepatic CCA (iCCA), perihilar CCA, and distal CCA.^1^ Patients not suitable for surgery receive systemic chemotherapy with gemcitabine and cisplatin in addition to standard care and have a median overall survival of <1 year.^2,3^ Recent research has shown that checkpoint inhibitors may be an emerging cornerstone to CCA therapy.^4^ Animal models of CCA allow us to study both the pathobiology of disease and treatment responses.^5^ The chemotoxic-induced model, responsible for inducing genotoxicity and promoting CCA formation, is a classic animal model of CCA.^6,7^ These chemotoxic substances include diethylnitrosamine (DEN) and N-nitrosodimethylamine (NDMA), nitrosamine, furan, thioacetamide (TAA), and carbon tetrachloride (CCL4).^5,6^ Previously, we reported that chronic cholestasis accelerated DEN-mediated CCA formation.^5^ Here, we established a novel mouse model recapitulating the progression from atypical cystic hyperplasia to CCA with DEN treatment.

Protein kinase CK2 (known as CSNK2) is a highly conserved serine/threonine kinase that regulates cell proliferation, cell cycle progression, invasiveness, and tumorigenesis.^8^ CSNK2α1 (CSNK2A1 and CK2α) plays a crucial role in cancer progression through MYC and Wnt/β-catenin pathways.^9^ Furthermore, CSNK2A1 can also induce phosphorylation of various molecules.^10^

β-catenin plays a crucial role in tumorigenesis as an intracellular signaling molecule in the WNT signaling pathway.^11^ High-mobility group box-1 (HMGB1) regulates apoptosis, autophagy, and gene transcription and is a critical protein in the pathogenesis of acute liver injury and chronic liver diseases.^12,13^ HMGB1 knockdown has been shown to inhibit proliferation and promote apoptosis and autophagy in CCA cell lines HuB28 and HuCCT1.^14^ It is established that proinflammatory cytokines, such as IL-6, enhance the pathogenesis of chronic inflammation-induced CCA. IL-6 released by cancer-associated fibroblasts has been shown to promote carcinogenesis.^15^ Elevated IL-6 has been considered a poor prognostic marker in patients with CCA.^15^

C-MYC is a basic helix-loop-helix leucine zipper transcription factor that forms a heterodimeric complex with MAX (MYC-associated factor X) and binds with the E-box sequence to activate transcription of target genes.^5^ We previously reported the importance of C-MYC in the murine model of CCA.^5^ It has been established that MAX acts as a tumor suppressor and rewires metabolism in small-cell lung cancer.^16^ Paradoxically, MAX deletion destabilizes MYC protein and abrogates Eµ-MYC lymphoma development.^17^ The role of MAX in CCA development remains largely unknown.

We developed a novel mouse model that progresses from atypical cystic hyperplasia to CCA. Compared with previously reported models, this model has a shorter CCA development time, more evident pathological manifestations, and a closer resemblance to the development of CCA in humans. Furthermore, using this model, we found that MAX and MAX-interacting proteins are targetable master regulators of CCA progression.

## METHODS

### Materials

N-nitrosodiethylamine (DEN, CAS Number: 55-18-5) and CX-4945 (Cat#: HY-50855B) were purchased from Sigma-Aldrich (St. Louis, MO) and Medchemexpress (Monmouth Junction, NJ), respectively. Other reagents were obtained from commercial sources.

### Additional methods

All other methods used are described in detail in the Supplemental Methods section of the Supplemental Materials.

## RESULTS

### Establishment of a novel model of cholangiocarcinogenesis

**FIGURE 1:**
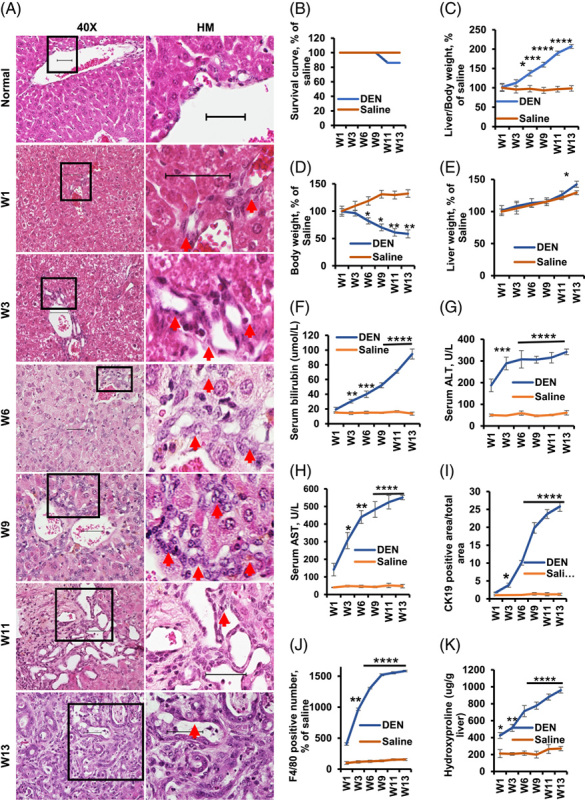
Pathological and biochemical changes in DEN-treated mice. (A) Representative livers showing cholangiocyte proliferation of bile duct in the portal area (weeks 1 and 3, denoted by arrow), atypical proliferation from portal areas to the periportal area along sinusoid and in the adjacent liver parenchyma with the formation of irregular and tortuous ductular structures (week 6, denoted by arrow), atypical cystic hyperplasia (week 9, denoted by arrow), cholangiomas showing neoplastic cells in small cords and the stroma extends around the reactive ductulus (week 11, denoted by arrow), and CCA showing the papillary proliferation of the atypical biliary epithelium with a multilayering of nuclei, loss of cell polarity, and nuclear hyperchromasia within the bile ducts (week 13, donated by arrow). Magnifications are ×20 for the images on the left; areas indicated by arrows are further magnified on the right for each. H&E representative pictures are shown from n=3. (B) Survival curves, % of saline in different groups of mice. Blue represents the DEN group and orange represents the control group. Liver/Body weight (C), body weight (D), and liver weight (E) of the mice, % of saline. Total serum was extracted from the treated groups to assess the serum bilirubin (μmol/L) (F), alanine aminotransferase (ALT, U/L) (G), and aspartate aminotransferase (AST, U/L) (H) levels. Analysis of CK19-positive area/total area (I), F4/80 positive number, % of saline (J), and hydroxyproline (ug/g liver) (K). Twelve samples from the DEN treatment groups and 6 samples from the control groups were used in the above assays. Control group vs. DEN treatment groups, *p < 0.05, **p < 0.01, ****p <0.001, and ****p <0.0001. Abbreviations: W1, week 1; W3, week 3; W6, week 6; W9, week 9; W11, week 11; W13, week 13.

Some chemically induced CCA models are suboptimal due to high mortality, low incidence, and the need for surgical procedures (Supplemental Table S1, http://links.lww.com/HC9/A362).^1,5^ At first, the effects of DEN treatment were assessed on biliary proliferation and development of atypical cystic hyperplasia, cholangiomas, and CCA. The intrahepatic bile ducts were confined to the portal space at week 1 (Figure 1A; Supplemental Figure S1A, http://links.lww.com/HC9/A350). Proliferating cholangiocytes formed a well-defined lumen at week 3 and resembled bile duct ligation at day 3 (Figure 1A and Supplemental Figure S1B, http://links.lww.com/HC9/A350). Biliary proliferation increased rapidly from weeks 3 to 6 (Figure 1A; Supplemental Figure S1C, http://links.lww.com/HC9/A350). Atypical cystic hyperplasia appeared 9 weeks after the administration of DEN. The proliferation of intrahepatic bile ducts extended to the periportal area along the sinusoid and adjacent liver parenchyma (Figure 1A; Supplemental Figure S2A, http://links.lww.com/HC9/A351) and instead were associated with inflammatory cell infiltration (Supplemental Figure S2B, http://links.lww.com/HC9/A351). The papillary proliferation of the atypical biliary epithelium showed multilayering of the nuclei, loss of cell polarity, and nuclear hyperchromasia within the bile ducts at week 13 (Figure 1A). Sinusoidal invasion and portal vein invasion are considered the most frequent modes of iCCA spread in humans; in our mouse model, we also found that tumor cells invaded sinusoids (Supplemental Figure S2C, http://links.lww.com/HC9/A351) and portal veins (Supplemental Figure S2D, http://links.lww.com/HC9/A351) at week 13. These pathological features demonstrate that the DEN-treated mouse model mimics human CCA progression well and is therefore an attractive model for studying biliary proliferation, atypical cystic hyperplasia, cholangiomas, and CCA progression.

Mice treated with DEN had 16% mortality at 11 and 13 weeks (Figure 1B). Significantly higher liver/body weight ratio (Figure 1C) and lower overall body weight (Figure 1D) were observed in the DEN-fed mice compared with control mice beginning 6 weeks after treatment. Significantly higher liver weights were found in the DEN-fed mice than in the control mice at week 13 (Figure 1E).

We examined changes in the hepatobiliary system over time after DEN treatment and found that bilirubin levels in mice treated with DEN increased sharply at week 3 and remained high up to week 13 (Figure 1F). An acute increase in alanine transaminase (ALT) and aspartate transaminase (AST) levels occurred after 1 week of oral gavage with DEN and steadily increased thereafter (Figure 1G, H).

CK19 was used to measure cholangiocyte proliferation. We found proliferating cholangiocytes (CK19 positive) at week 1 that increased dramatically from weeks 3 to 9 and plateaued around weeks 11 and 13 in the DEN group (Figure 1I; Supplemental Figure S3A, http://links.lww.com/HC9/A352). Hepatic macrophages are often referred to as KCs, since KCs represent the main fraction of liver macrophages. Mouse KCs are identified by F4/80 staining. We observed a significant increase in the number of F4/80-positive cells in mice livers treated with DEN at week 1, and levels continued to increase up to week 13 (Figure 1J; Supplemental Figure S3B, http://links.lww.com/HC9/A352).

Biliary proliferation and ductal fibrosis are commonly seen in CCA. alpha-smooth muscle actin (α-SMA)-positive staining increased gradually from week 1 to week 13 in mice livers treated with DEN (Supplemental Figure S3C, http://links.lww.com/HC9/A352). Proliferating cell nuclear Ag (PCNA) staining was used to measure cholangiocyte proliferation.^5^ We found PCNA-positive cholangiocytes gradually increased from week 1 to week 13 after treatment with DEN (Supplemental Figure S4A, http://links.lww.com/HC9/A353). We also detected the tumor malignity by carbohydrate Ag 19-9 (CA19-9) and carcinoembryonic Ag (CEA). Interestingly, intense cytoplasmic staining for CA19-9 (Supplemental Figure S4B, http://links.lww.com/HC9/A353) and CEA (Supplemental Figure S4C, http://links.lww.com/HC9/A353) was found in liver tissues with CCA. Sirius Red staining showed that collagen was present around the portal tracts and increased steadily from week 1 to week 13 (Supplemental Figure S6A, http://links.lww.com/HC9/A355). Cholangiomas showed the proliferating biliary structures are lined by a single layer of relatively uniform cuboidal epithelium (Supplemental Figure S5A, http://links.lww.com/HC9/A354). The discrete nodular growths were comprised of large acini lined by cuboidal epithelium with occasional papillary projections into the lumen (Supplemental Figure S5A, http://links.lww.com/HC9/A354). This cholangiomas has connective tissue stroma between acini (Supplemental Figure S5A, http://links.lww.com/HC9/A354). Cholangiocarcinoma showed the nuclear crowding (Supplemental Figure S5B, http://links.lww.com/HC9/A354). The tumors are composed of cuboidal to low columnar tumor cells (Supplemental Figure S5B, http://links.lww.com/HC9/A354). The liver hydroxyproline content (Figure 1K) increased beginning after 1 week in the DEN-treated group. Atypical cystic hyperplasia was observed in 83% of mice. The incidence rates of cholangiomas by weeks 11 and 13 were 66% and 75%, respectively. The incidence rate of CCA was 8% at week 11 and 33% at week 13 (Supplemental Figure S6B, http://links.lww.com/HC9/A355). The incidence rates of small nodular hepatocellular hyperplasia by weeks 11 and 13 were 8% and 16%, respectively (Supplemental Figure S6B, C, http://links.lww.com/HC9/A355). The combined incidence rates of small nodular cholangiocellular and hepatocellular hyperplasia were 8% for week 11 and 16% for week 13 (Supplemental Figure S6B, D, http://links.lww.com/HC9/A355).

### MAX expression and effect on cell growth, migration, invasion, and tumorigenesis

**FIGURE 2:**
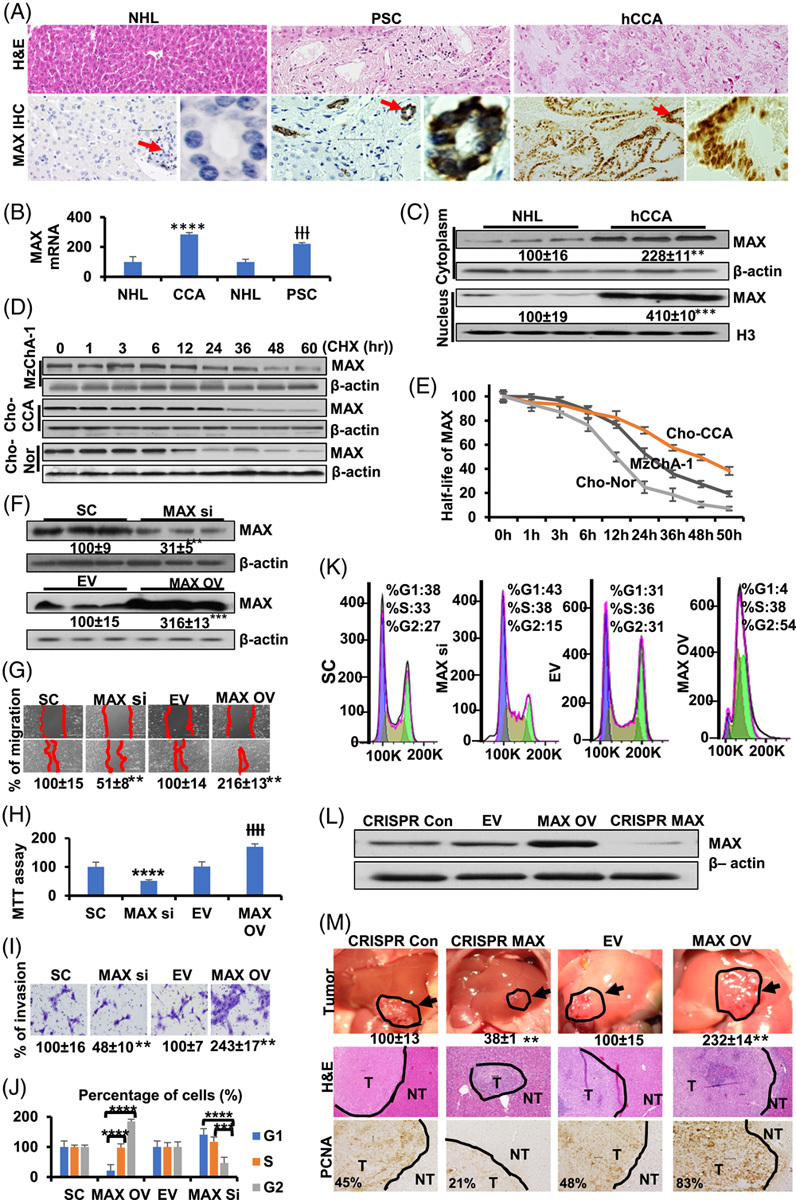
Expression and stability of MAX and its role in MzChA-1 growth, migration, invasion, and tumorigenicity. (A) MAX strongly stained in the proliferated bile duct of primary sclerosing cholangitis (PSC) and cholangiocarcinoma (CCA). H&E stains are shown in the top (×10), and IHCs are shown in the bottom (×20). Local magnification of boxed areas is shown right for each IHC. IHC representative pictures are shown from n=3. (B) MAX mRNA levels in NHL, CCA and PSC from 2-way ANOVA assay. Results are mean % ± SEM of NHL. †††*p* < 0.001, ****p  <  0.0001 vs. NHL. (C) MAX protein level in cytoplasm and nucleus from NHL and CCA was measured by Western blotting (n=3 each) were normalized to housekeeping control β-actin (cytoplasm) or nucleus (Histone H3). Densitometric values are summarized below the blots, expressed as mean% of normal liver ± SEM. **p  <  0.01, ***P<0.001 vs. normal liver. (D) MzChA-1 and cholangiocytes isolated from mouse liver tissues with CCA and control livers were cultured at 24 hours, and then CHX treatment and Western blotting (top) were conducted. The MAX band intensity was normalized to β-actin and then normalized to the t=0 controls. Graph showing relative level of MAX below western blotting. Data are shown as mean ± SEM, in 3 different experiments (n=3) (bottom). (E) The half-life of MAX in cholangiocytes from CCA (above) and normal tissue (mid) and MzChA-1 (below), *p <0.05 in 2-way ANOVA assays. (F) Protein levels of MAX were measured in MzChA-1 and normalized to housekeeping β-actin to determine the efficiency of MAX silence (MAX Si) and overexpression (MAX OV). Numbers below the blots represent mean ± SEM densitometric changes, expressed as % of scramble siRNA (SC) or empty vector (EV). ** *p* <0.01, *** *p* <0.001. Data are shown as mean ± SEM. in 3 different experiments by 2-way ANOVA assays (n=3). (G–I) MzChA-1 cells were transfected with MAX siRNA, MAX overexpressed vector, SC, or EV for 24 hours. Results are expressed as mean % of control ± SEM from 3 experiments done, ** *p* <0.01, ***p <0.001 vs. SC or EV. Migration (G), MTT (H), and invasion (I) assays were done in MzChA-1 cells after treatment with MAX siRNA or MAX overexpressed vector for 24 hours. Results are shown as mean% of SC or EV ± SEM from 3 independent experiments done. MTT statistic assays were done by 2-way ANOVA. **p <0.01, ****p  < 0.0001 vs. SC or EV. ††††P<0.0001. (J and K) The effect of MAX OV or siRNA on cell cycle progression in the MzChA-1 cell line. (J) Distribution of the MzChA-1 cells in different phases of the cell cycle were determined by flow cytometry and analyzed with FCS Express 5 Flow software to determine the percentage of cells in each phase of the cell cycle. (K) Flow cytometry was done for fraction of MzChA-1 cells in the G1, S, and G2 phases in MAX OV or siRNA. **** *p* <0.0001 G1 or S phase vs. G2 phase. (L) Protein levels of MAX OV and CRISPR MAX were measured in the MzChA-1 cell. (M) Morphological changes of liver after CRISPR MAX and MAX OV treatment. Arrow points to tumor (top). H&E staining is shown in the middle, and PCNA staining is shown in the bottom. PCNA-positive cells are shown as mean % in tumor tissues. Abbreviations: CHX, cycloheximide; Chol-CCA, cholangiocytes from CCA liver tissues; Chol-Nor, cholangiocytes from normal liver tissues; eCCA, extrahepatic CCA; EV, empty vector; iCCA, intrahepatic CCA; NBEC, normal biliary epithelial cells; NHL, normal human liver; N, sample number; NT, nontumor bile duct; NTBD, nontumor bile duct; OV, overexpressed; SC, scramble siRNA.

MAX has both favorable and unfavorable consequences in tumorigenesis.^16,18–20^ We focused on MAX because we found higher human MAX protein and mRNA levels in PSC and CCA compared with normal liver, especially in the nucleus of human and mouse CCA (Figure 2A, B and Supplemental Figure S7A, B, http://links.lww.com/HC9/A356). MAX mRNA levels are higher in iCCA compared with adjacent nontumor liver tissue (GSE76297) (Supplemental Figure S8A, http://links.lww.com/HC9/A357) and iCCA compared with normal human biliary epithelial cells (GSE32225) (Supplemental Figure S8B, http://links.lww.com/HC9/A357). Moreover, the GEPIA database shows that MAX mRNA significantly increases in CCA compared with normal liver tissues (Supplemental Figure S8C, http://links.lww.com/HC9/A357). MAX protein levels in the cytoplasm and nucleus were higher in human CCA compared with normal liver tissues (Figure 2C). One possible explanation is that MAX affects cellular stability. To investigate this possibility, we performed a chase assay using cycloheximide and observed that the half-life of MAX was ~24 hours in MzChA-1 cells, 48 hours in cholangiocytes isolated from mouse liver with CCA, and 12 hours in cholangiocytes isolated from control mice (Figure 2D, E). Therefore, the stability of MAX from normal cholangiocytes was lower than that of MzChA-1- and CCA-derived cholangiocytes (Figure 2E). The enhanced stability of MAX in cancer cells led us to examine whether it regulated cell proliferation. Compared with scramble siRNA, MAX siRNA inhibited MAX protein by 69% (Figure 2F). In contrast, overexpression of MAX led to a 2.2-fold increase in its protein level (Figure 2F). We found that silencing MAX suppressed MzChA-1 migration (Figure 2G), cell growth (Figure 2H), and invasion (Figure 2I).

MAX knockdown of MzChA-1 cells increased G1 fraction and decreased the G2 fraction relative to cells transduced with control siRNA (Figure 2J, K), indicating inhibited cell cycle progression. MAX overexpression in MzChA-1 cells decreased G1 fraction and increased the G2 fraction relative to cells transduced with empty vector (Figure 2J, K), indicating promoted cell cycle progression. Overexpression of MAX had a profound promotion of anchorage-independent growth, whereas MAX silencing has the opposite effect (Supplemental Figure S9, http://links.lww.com/HC9/A358).

To confirm their effects in vivo, we established MzChA-1 cell lines that either stably overexpress MAX or have reduced expression of MAX using CRISPR as described^21^ (Figure 2L). These cells were implanted into the left lobe of the liver, and their growth was examined in this orthotopic model. Figure 2M (top) shows the tumor at the site of injection after 30 days. CCA cells overexpressing MAX resulted in much larger tumor sizes as compared with respective controls. In contrast, CCA cells overexpressing CRISPR targeting MAX had much smaller tumor sizes (Figure 2M). Figure 2M (middle) shows H&E staining of the tumors and the aggressive histological features of the MAX overexpressing or the MAX knocked‐down CCAs. Consistently, PCNA staining is highest for tumor overexpressing MAX, and the opposite is true for MAX knockdown (Figure 2M, bottom).

### HMGB1, WNT5B, C-MYC, β-catenin, and IL-6 are interacting proteins of MAX in CCA

**FIGURE 3:**
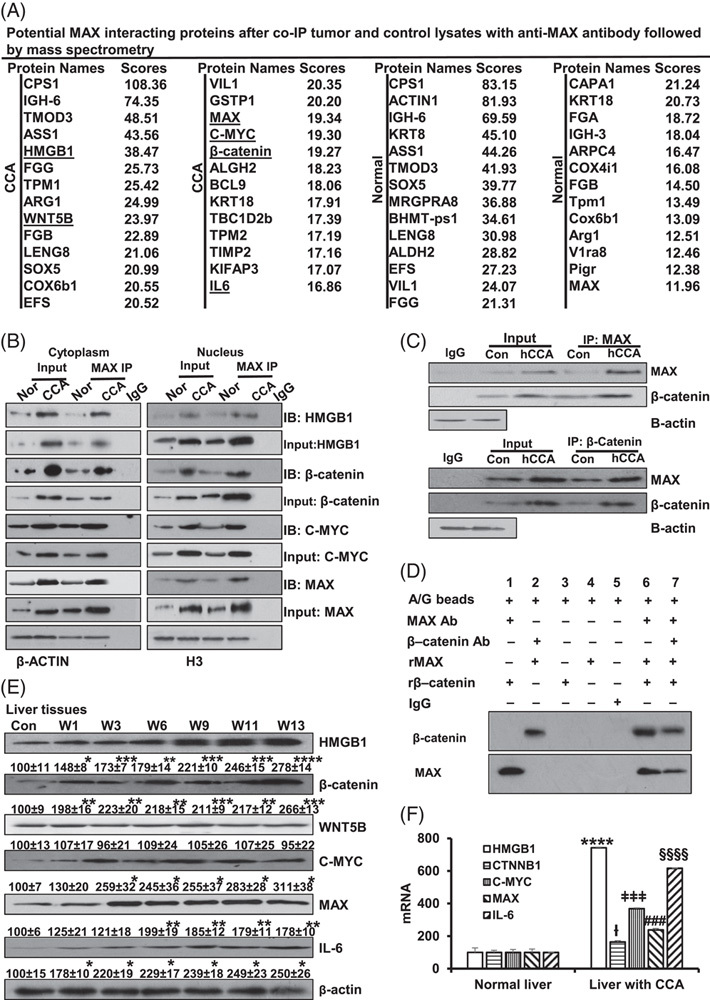
The analysis of MAX and potential MAX-interacting proteins. (A) Potential MAX-interacting proteins after coimmunoprecipitation (Co-IP) of CCA and normal lysates with anti-MAX antibody followed by mass spectrometry. Three specimens per group were pooled for the IP. (B) Verification of potential MAX-interacting proteins in the cytoplasm and nucleus isolated from liver tissues with CCA and normal control were detected by MAX IP and western blotting. (C) MAX and β-catenin interaction in Con and hCCA were detected by Co-IP and western blotting. (D) In vitro pull-down shows direct interaction between MAX and β-catenin using recombinant MAX and β-catenin proteins. Results represent 3 independent experiments done in duplicate. (E) Western blots show the time course of protein expression of HMGB1, β-catenin, WNT5B, C-MYC, MAX, and IL-6 in liver tissue. Densitometric values are shown below the blots, expressed as mean% of Con ± SEM from 3 experiments done, *p  < 0,05. **p  < 0.01, ***p  < 0.001, ****p  < 0.0001 vs. Con. (F) The mRNA level of HMGB1, CTNNB1, C-MYC, MAX, and IL-6 isolated from livers between CCA and normal controls. Abbreviations: ACT1, actin1 gene; ALDH2, aldehyde dehydrogenase 2; Arg1, arginase1; ARPC4, actin related protein 2/3 complex subunit 4; ASS1, argininosuccinate synthetase; BCL9, B-cell lymphoma 9; Bhmt-ps1, betaine-homocysteine methyltransferase, pseudogene 1; CAPA1, calpain 1; COX4I1, cytochrome c oxidase subunit 4I1; COX6B1, cytochrome c oxidase subunit 6B1; CPS1, carbamoyl phosphate synthetase 1; EFS, embryonal Fyn-associated substrate; FGA, fibrinogen alpha chain; FGB, fibrinogen beta chain; FGG, fibrinogen gamma chain; GSTP1, Glutathione S-transferase Pi; hCCA, hilar cholangiocarcinoma; KIFAP3, KIF-associated protein 3; KRT18, keratin 18; IGH3(Ighg2b), immunoglobulin heavy constant gamma 2B; LENG8, leukocyte receptor cluster, member 8; Mrgpra8, MAS-related GPR member A8; PIGR, polymeric immunoglobulin receptor; SOX5, SRY-box transcription factor 5; TMOD3, tropomodulin 3; TPM1, Tropomyosin alpha-1 chain; VIL1, Villin 1; V1ra8, vomeronasal 1 receptor, A8.

To define the MAX interactome, we performed immunoprecipitation (IP) with an anti-MAX antibody using liver lysates from CCA and saline control mice. Proteins were identified using mass spectrometry (Figure 3A).

Co-IP of HMGB1, WNT5B, C-MYC, β-catenin, and IL-6 with MAX was enhanced in CCA liver tissues compared with controls (Figure 3A, Supplemental Tables S2, http://links.lww.com/HC9/A363 S3, http://links.lww.com/HC9/A364). By bioinformatics analysis, we found these proteins to be correlated with cancer pathways. The correlation of these proteins with cancer pathways was evaluated using DAVID Bioinformatics Resources (https://davidncifcrf.gov), KEGG pathway, and GO terms. C-MYC, CTNNB1, and IL-6 were strongly associated with “pathways of cancer” (Supplemental Figure S10A, http://links.lww.com/HC9/A359). MYC and CTNNB1 were also linked to “pathways in tumor progression” (Supplemental Figure S10B, http://links.lww.com/HC9/A359). HMGB1 was involved in “positive regulation of cell migration and invasion” (Supplemental Figure S10C, http://links.lww.com/HC9/A359). Meanwhile, C-MYC and CTNNB1 were the most relevant MAX-interacting proteins in “proteoglycans in cancer” pathways in CCA, with no reference in normal tissues (Supplemental Figure S10D, http://links.lww.com/HC9/A359). Some proteins such as Bcl9, TBC1d2b, and kifap3 also had higher scores as MAX-interacting proteins but were not related to cancer pathways. Hence, HMGB1, WNT5B, C-MYC, β-catenin, and IL-6 were considered as possible important MAX-interacting proteins in CCA by bioinformatics analysis. Furthermore, analysis of these partners in GEPIA human CCA database showed that IL-6, CTNNB1, HMGB1, and C-MYC exhibited a positive correlation with MAX mRNA levels (Supplemental Figure S10E–H, http://links.lww.com/HC9/A359).

To validate these novel MAX interactors, we performed Co-IP with an anti-MAX antibody followed by western blotting from CCA and normal mouse liver lysates. HMGB1, β-catenin, and C-MYC interacted with MAX in the cytoplasm and nucleus (Figure 3B). We also performed Co-IP with human CCA and normal human liver lysates and found higher levels of MAX interaction and more interaction with β-catenin in the cancer tissues (Figure 3C). We next used recombinant MAX, β-catenin, and specific antibodies immobilized to A/G beads and found MAX and β-catenin can interact directly (Figure 3D). We also found that DEN treatment increased HMGB1, β-catenin, C-MYC, and IL-6 protein expression (Figure 3E) and mRNA levels (Figure 3F) compared with control.

### Cell-specific expression of MAX and MAX-interacting proteins in liver tissues with CCA

**FIGURE 4:**
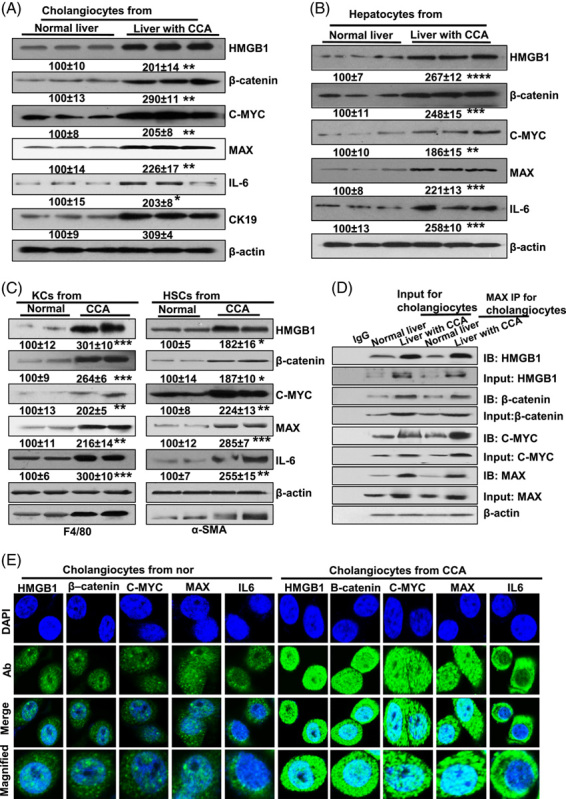
The cell-specific expression of MAX and MAX-interaction proteins, and verification of interaction and cellular location. The protein of HMGB1, β-catenin, C-MYC, MAX, CK19, and IL-6 in cholangiocytes (A). The protein of HMGB1, β-catenin, C-MYC, MAX, and IL-6 in hepatocytes (B), KCs (C, left) or HSCs (C, right) isolated from livers with CCA and normal controls. (D) Verification for HMGB1, β-catenin, C-MYC and MAX interaction for cholangiocytes isolated from CCA and control liver was detected by Co-IP and western blotting. (E) Immunofluorescence (IF) of cholangiocytes from normal and CCA liver after transfections of HMGB1, β-catenin, C-MYC, MAX, and IL-6. The top row shows DAPI staining. The second row shows the Ab staining. The third row merged DAPI, and Ab staining [original magnification, ×630 (oil immersion)] and the fourth row shows the magnified images of the regions. Abbreviations: Ab, antibodies; H69, human normal cholangiocyte H69 cells; HuCCT1, human intrahepatic CCA cells; MzChA-1, human biliary adenocarcinoma.

To determine the expression levels of MAX and MAX-interacting proteins, hepatocytes, KCs, HSCs, and cholangiocytes were isolated from liver with CCA and normal control. We found that the protein expression of MAX and HMGB1, β-catenin, C-MYC, and IL-6 was elevated in cholangiocytes from CCA tissue compared with control (Figure 4A). We further isolated hepatocytes from mouse control and CCA livers and assessed the expression of MAX and its interacting proteins. The protein expression of HMGB1, β-catenin, C-MYC, and IL-6 in CCA-derived hepatocytes was 2.67-, 2.48-, 1.86-, and 2.58-fold, respectively, compared with control group (Figure 4B).

HMGB1 in KCs isolated from CCA liver tissues was 2-fold higher than controls (Figure 4C, left). Our results also showed that the protein expression of β-catenin was 1.64-fold higher in the CCA-derived KCs compared with the control group (Figure 4C, left). IL-6 expression in KCs isolated from CCA livers was 2.0-fold higher than that of control mice.

It is well known that activated HSCs promote cancer cell progression through paracrine or autocrine IL-6 signaling.^22^ Our results showed that IL-6 levels were increased 1.55-fold in HSCs isolated from CCA liver tissues compared with the control group. HMGB1 expression in HSCs isolated from CCA liver tissues was increased 1.82-fold, while C-MYC and MAX were increased 2.24 and 1.85-fold, respectively, in the CCA group compared with controls (Figure 4C, right). Cholangiocytes derived from CCA liver exhibited increased interaction of HMGB1, β-catenin, and C-MYC with MAX compared with control cholangiocytes (Figure 4D). Furthermore, immunofluorescence showed that the expression of HMGB1, C-MYC, and β-catenin in cholangiocytes from CCA liver was higher than normal liver in the cytoplasm and nucleus while IL-6 increased just in cytoplasm (Figure 4E).

### CSNK2A1-mediated MAX phosphorylation increased C-MYC and β-catenin binding and regulated HMGB1 promoter activity through E-BOX

**FIGURE 5:**
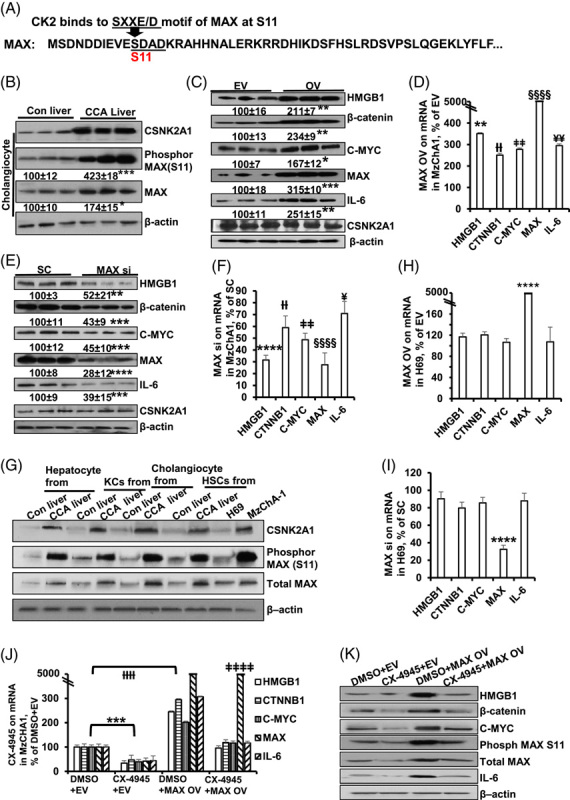
CSNK2A1, CX-4945 and MAX phosphorylation, and MAX’s effect on MAX-interaction proteins. (A) MAX contains an important binding site at S11. (B) Phospho-MAX (S11) protein levels in the cholangiocytes isolated from mouse liver tissues with CCA and normal livers. Densitometric values are summarized below the blots, expressed as mean% of normal liver ± SEM. *p  < 0.05, ***P<0.001 vs. cholangiocytes from normal liver. (C, D) The mRNA and protein levels of HMGB1, β-catenin, C-MYC, MAX, and IL-6 after MAX OV in MzChA-1 cells. (E, F) The mRNA and protein levels of HMGB1, β-catenin, C-MYC, MAX, and IL-6 after MAX siRNA treatment in MzChA-1 cells. (G) Protein expression patterns of CSNK2A1, phosphor S11 MAX, and MAX in hepatic cells, H69, and MzChA-1. (H, I) The mRNA levels of HMGB1, β-catenin, C-MYC, MAX, and IL-6 after MAX knockdown (H) and overexpression (I) in H69, % of SC or EV. (J, K) The mRNA and protein levels of HMGB1, β-catenin, C-MYC, MAX, and IL-6 after DMSO+EV, CX-4945+EV, DMSO+MAX OV, and CX-4945+MAX OV in MzCHA-1. For mRNA, results are mean % ± SEM of SC or EV. **p  < 0.01, ****p  < 0.0001 vs. SC or EV for HMGB1. ††*p* <0.01, ††† *p* <0.001 vs. SC or EV for CTNNB1. ‡‡*p*  < 0.01, ‡‡‡*p*  < 0.001, ‡‡‡‡*p*  < 0.0001 vs. SC for C-MYC. §§§*p*<0.001, §§§§*p*<0.0001 vs. SC or EV for MAX. ¥*p*<0.05, ¥¥*p*<0.01, ¥¥¥*p*<0.001, ¥¥¥¥*p*<0.0001 vs. SC for IL-6. Densitometric values are summarized below the western blots, expressed as mean% of normal liver ± SEM. ** *p*<0.01, *****p<0.001, ****p<0.0001 vs. SC or EV. Data are shown in 3 different experiments (n=3).

CK2α inhibits the DNA-binding activity of MAX-MAX homodimers but not MYC-MAX heterodimers.^23^ CK2/CSNK2A1 is involved in cancer progression by phosphorylating various signaling molecules.^9^ We therefore hypothesized that the switch of MAX-MAX homodimerization to MYC-MAX or β-catenin-MAX heterodimerization may regulate HMGB1 promoter activity through the E-box. MAX contains one important canonical SXXE/D motif of CK2^24^ at S11. CSNK2A1 was predicted to bind the SXXE/D motif of MAX at the S11 phospho-site (Figure 5A). We also found that CSNK2A1 and MAX S11 expression was increased in cholangiocytes from liver tissue with CCA compared with controls (Figure 5B). Available CCA data sets showed that CSNK2A1 mRNA levels are higher in iCCA compared with adjacent nontumor liver tissue (GSE76297) (Supplemental Figure S8F, http://links.lww.com/HC9/A357) and iCCA compared with normal human biliary epithelial cells (GSE32225) (Supplemental Figure S8G, http://links.lww.com/HC9/A357). Moreover, the GEPIA database shows that CSNK2A1 mRNA significantly increases in CCA compared with normal liver tissues (Supplemental Figure S8D, http://links.lww.com/HC9/A357), and the mRNA of CSNK2A1 and MAX are positively correlated with each other (Supplemental Figure S8E, http://links.lww.com/HC9/A357). Even though overexpression or knockdown of MAX positively or negatively regulated HMGB1, β-catenin, C-MYC, and IL-6 protein expression and mRNA levels in MzChA-1 (Figure 5C–F) and HuCCT1 (Supplemental Figure S11A–D, http://links.lww.com/HC9/A360), MAX modulation did not alter CSNK2A1 protein expression (Figure 5C, E). Compared with MzChA-1 cells that had high CSNK2A1 levels, H69 cells have very low CSKN2A1 expression (Figure 5G). We found that the expression of HMGB1, β-catenin, C-MYC, and IL-6 in H69 cell line could not be altered by overexpressing or silencing MAX (Figure 5H, I). The overall data indicate that MAX’s effects on C-MYC, β-catenin, HMGB1, and IL-6 may be dependent on the cellular level of CSNK2A1.

To further assess the role of CSNK2A in regulating MAX’s function, we used the specific and selective CK2 ATP competitive inhibitor (CX-4945, Silmitasertib) that has been administered in human trials as an anticancer drug. We found that CX-4945 could inhibit mRNA and protein expression of basal and MAX OV-induced C-MYC, β-catenin, HMGB1, and IL-6 in MzChA-1 (Figure 5J, K). Since there is low expression of CSNK2A1 and phospho-max in H69 (Figure 5G), it is possible that CX-4945 may not work there in inhibiting MAX targets (Supplemental Figure S10E, F, http://links.lww.com/HC9/A359).

### MAX phosphorylation is involved in upregulation of HMGB1 and IL-6 promoter activity

**FIGURE 6:**
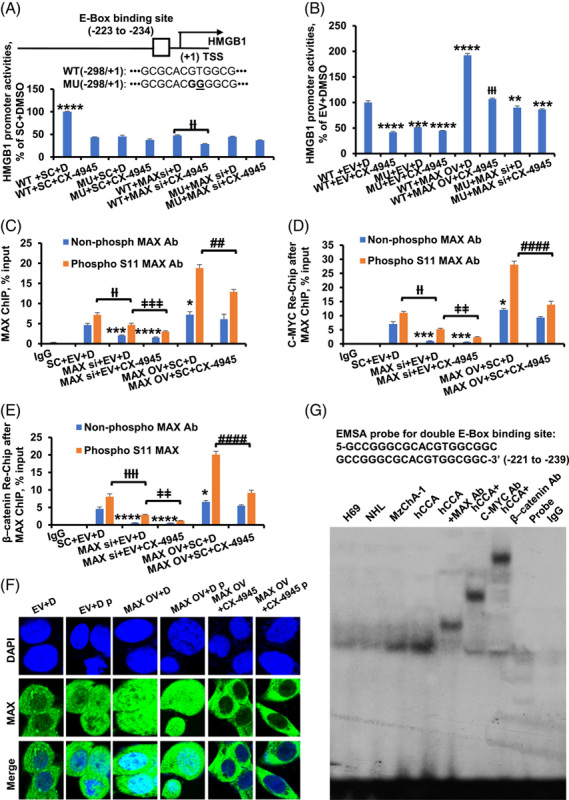
CSNK2A1- and CX-4945-mediated MAX phosphorylation regulate HMGB1 promoter activity through E-box element. (A) MAX siRNA and CX-4945 regulated HMGB1 promoter activity in MzChA-1 cells. Results are mean % of WT+SC+D ± SEM from 3 experiments done, ****p  < 0.0001 vs. others, ††*p* <0.01.vs. WT+SC+D. The top diagram shows E-box element and its mutant in the human HMGB1 promoter. (B) MAX overexpression and CX-4945 regulated HMGB1 promoter activity in MzChA-1 cells. Results are mean % of WT+EV+D ± SEM from 3 experiments done, **p <0.01, ***p <0.001, ****p  < 0.0001, †††*p* <0.001 vs. WT+MAX OV+D. (C) ChIP and qPCR analysis was performed by spanning E-box region of the HMGB1 promoter in MzChA-1 cells using MAX and phosphor S11 MAX antibodies. Results are mean % of input ± SEM from 3 experiments done, *p <0.05, ***p <0.001, ****p <0.0001 vs. SC+EV+D in MAX antibody, ††*p*<0.01 SC+EV+D vs. MAX si+EV+D, ‡‡‡*p* <0.001 MAX si+EV+D vs. MAX si+EV+CX-4945, ##*p*  < 0.01 MAX OV+SC+D vs. MAX OV+SC+CX-4945 in phosphor S11 MAX antibody. (D) Seq-ChIP and qPCR analysis was performed by spanning E-box region of the HMGB1 promoter in MzChA-1 cells using C-MYC antibody after ChIP of MAX and phosphor S11 MAX antibodies. Results are mean % of input ± SEM from 3 experiments done, *p <0.05, ***p <0.001 vs. SC+EV+D in MAX antibody, ††*p*<0.01 SC+EV+D vs. MAX si+EV+D, ‡‡*p* <0.01 MAX si+EV+D vs. MAX si+EV+CX-4945, ####*p*  < 0.0001 MAX OV+SC+D vs. MAX OV+SC+CX-4945 in phosphor S11 antibody. (E) Seq-ChIP and qPCR analysis was performed by spanning E-box region of the HMGB1 promoter in MzChA-1 cells using β–catenin antibody after ChIP of MAX and phosphor S11 MAX antibodies. Results are mean % of input ± SEM from 3 experiments done, *p <0.05, ****p <0.001 vs. SC+EV+D in MAX antibody, ††††*p*<0.0001 SC+EV+D vs. MAX si+EV+D, ‡‡*p* <0.01 MAX si+EV+D vs. MAX si+EV+CX-4945, ####*p*  <  0.0001 MAX OV+SC+D vs. MAX OV+SC+CX-4945 in phosphor S11 antibody. (F) IF of MAX in cholangiocyte after transfections. The top row shows DAPI staining. The second row shows the MAX staining [including antibodies of MAX and phosphor S11 MAX (*p*)]. The third row merged DAPI and MAX staining [original magnification, ×630 (oil immersion)]. (G) EMSA analyses using labeled probes containing 2 E-box elements of the HMGB1 promoter were performed in H69 and MzChA-1 cells. Probe only and IgG served as controls. Abbreviations: Ab, antibody; D, DMSO; EV, empty vector; MU, E-box element CACGTG mutation into CACGGG in −298/+1 HMGB1 promoter; *p*, phospho S11 MAX antibody; SC, scramble siRNA; Seq-ChIP, sequencing chromatin immunoprecipitation; WT, −298/+1 wild-type HMGB1.

The HMGB1 promoter region (−298/+1) (Supplemental Figure S12, http://links.lww.com/HC9/A361) was assessed for its responsiveness to MAX overexpression in MzChA-1 cells. MAX knockdown lowered HMGB1 promoter-driven luciferase activity, whereas MAX OV had the reverse effect (Figure 6A, B). To evaluate whether the effect of MAX on the HMGB1 promoter was through E-box region, we mutated the E-box-binding site in the promoter. Mutation at the E-box-binding site lowered HMGB1 promoter activity by about 50% (Figure 6A, B). CX-4945 decreased HMGB1 promoter activity, but this was attenuated with E-box mutation (Figure 6A, B). To determine whether phosphorylation of MAX is involved in HMGB1 promoter activities, ChIP and qPCR analysis were performed by spanning the E-box region in MzChA-1 cells using MAX and phospho S11 MAX antibodies. ChIP assays showed MAX knockdown lowered MAX binding to E-box region of the HMGB1 promoter. MAX overexpression increased MAX binding to E-Box region. There was more binding to the E-box in phosphorous S11 MAX compared with nonphosphorous MAX. Interestingly, CX-4945 treatments enhanced the effect of MAX siRNA knockdown and overexpression (Figure 6C). It explains MAX phosphorylation mediates HMGB1 promoter activities.

MAX phosphorylation may increase combination of phosphorylated MAX with C-MYC or β-catenin. Seq-ChIP and qPCR analysis was performed using C-MYC antibody after ChIP of MAX and phospho S11 MAX antibodies. Results showed that MAX knockdown or overexpress lowered or increased C-MYC binding to E-box region of the HMGB1 promoter, respectively. Furthermore, there was more binding to the E-box in phosphorous S11 MAX compared with nonphosphorous MAX. Moreover, CX-4945 treatments enhanced the effect of MAX siRNA knockdown and overexpression (Figure 6D, E). Immunofluorescence in cholangiocytes after MAX OV treatments showed that MAX staining (including antibodies of MAX and phospho S11 MAX) increases in cytoplasm and nucleus. Phospho-MAX staining of nucleus increases markedly in MAX OV+ phosphor S11 MAX antibody compared to MAX OV+ MAX antibody. CX-4945 treatment inhibits nuclear immunofluorescence staining of MAX (Figure 6F). Intrigued by the MAX-, C-MYC-, and β-catenin–mediated regulation of HMGB1 reporter activity, we examined whether MAX, C-MYC, and β-catenin can bind to the E-box element using nuclear proteins from H69, normal human liver, MzChA-1, and human CCA liver. Figure 6G shows that MAX, C-MYC, and β-catenin can bind to the E-box element (Shift bands, Figure 6G). Interestingly, β-catenin can heterodimerize with MAX to bind to the E-box element (Figure 6G). This suggests that MAX’s inductive effect may be, in part, by increasing β-catenin’s expression and binding to the E-box element. Meanwhile, these results suggested that phospho S11 MAX may positively regulate the promoter activities by interacting with and enhancing the binding of C-MYC and β-catenin to the E-box element, which positively regulated HMGB1 transcription.

**FIGURE 7:**
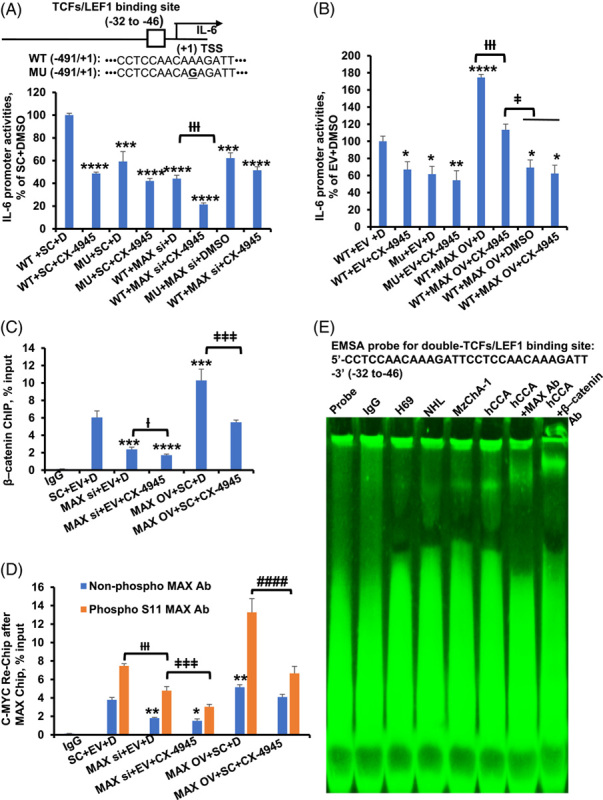
CSNK2A1 and CX-4945-mediated MAX phosphorylation regulate IL-6 promoter activity through TCF1/LEF1 element. (A) MAX siRNA and CX-4945 regulated IL-6 promoter activity in MzChA-1 cells. Results are mean % of WT+SC+D ± SEM from 3 experiments done, ***p <0.001, ****p  < 0.0001 vs. others, †††*p* <0.001.vs. WT+SC+D. The top diagram shows TCFs/LEF1 element and its mutant in the human IL-6 promoter. (B) MAX overexpression and CX-4945 regulated IL-6 promoter activity in MzChA-1 cells. Results are mean % of WT+EV+D ± SEM from 3 experiments done, *p <0.05, **p <0.01, ****p  < 0.0001 vs. WT+EV+D, †††*p* <0.001 vs. WT+MAX OV+D, ‡*p* <0.05 vs. WT+MAX OV+CX-4945. (C) ChIP and qPCR analysis was performed by spanning TCFs/LEF1 region of the IL-6 promoter in MzChA-1 cells using β-catenin antibody. Results are mean % of input ± SEM from 3 experiments done, ***p <0.001 vs. SC+EV+D in β-catenin antibody, †*p*<0.05 vs. MAX si+EV+D, ‡‡‡*p* <0.001 vs. MAX OV+SC+D. (D) Seq-ChIP and qPCR analysis was performed by spanning TCFs/LEF1 region of the IL-6 promoter in MzChA-1 cells using MAX and phosphor S11 MAX antibodies after β-catenin antibody ChIP. Results are mean % of input ± SEM from 3 experiments done, *p <0.05, **p <0.001 vs. SC+EV+D in MAX antibody, †††*p*<0.01 vs. SC+EV+D, ‡‡‡*p* <0.01 vs. MAX si+SC+D in phosphor S11 antibody. #### *p* <0.0001 vs. MAX OV+SC+D in phosphor S11 antibody (E) EMSA analyses using probe containing double TCFs/LEF1 element of the IL-6 promoter were performed in H69 and MzChA-1 cells. Probe only and IgG served as controls. Abbreviations: Ab, antibody; D, DMSO; EV, empty vector; MU, TCFs/LEF1 element CCTCCAACAAAGATT mutation to CCTCCAACAGAGATT in −491/+1; SC, scramble siRNA; Seq-ChIP, sequencing chromatin immunoprecipitation; WT, −491/+1 wild-type IL-6 promoter.

Furthermore, we also assessed IL-6 promoter activities using MAX knockdown and overexpression in MzChA-1 cells. MAX knockdown and overexpression decreased and increased IL-6 promoter-driven luciferase activity, respectively (Figure 7A, B). To explore the regulatory site of MAX on the IL-6 promoter, we mutated the TCFs/LEF1-binding site in the IL-6 promoter. Mutations in the TCFs/LEF1-binding site and CX-4945 both reduced IL-6 promoter activity. Interestingly, the effect of CX-4945 on reducing IL-6 promoter activity was attenuated by TCFs/LEF1 mutations (Figure 7A, B). However, it was uncertain whether the phosphorylation of MAX was involved in the regulation of IL-6 promoter activity. We performed Seq-ChIP assays using MAX and phosphor S11 MAX antibodies after β-catenin ChIP by spanning the TCFs/LEF1 region in MzChA-1 cells. The results showed that MAX knockdown and CX-4945 reduced the binding of β-catenin binding to the TCFs/LEF1 region of the IL-6 promoter, whereas MAX OV had the opposite effect. CX-4945 reduced MAX OV-mediated IL-6 promoter activities (Figure 7C). To determine whether MAX phosphorylation increases β-catenin binding to TEFs/LCF1 site, ChIP, Seq-ChIP and qPCR were performed using MAX and phospho S11 MAX antibodies. Phospho-MAX staining of nucleus increases markedly in MAX OV+ phosphor S11 MAX antibody compared to MAX OV+ MAX antibody. CX-4945 treatment inhibits nuclear immunofluorescence staining of MAX (Figure 6F). The results showed that MAX knockdown or overexpression decreased or increased the binding of MAX to the IL-6 promoter TCFs/LEF1 region, respectively. In addition, there was more binding to the E-box in phosphorus S11 MAX compared with nonphosphorus MAX. CX-4945 treatment enhanced the effect of MAX siRNA knockdown and overexpression (Figure 7D). Impressed by the MAX and β-catenin–mediated regulation of IL-6 reporter activity, we examined whether MAX and β-catenin can bind to the TEFs/LCF1 element using nuclear proteins from H69, normal human liver, MzChA-1, and human CCA liver. Figure 7E shows that MAX and β-catenin can bind to the TEFs/LCF1 element (Shift bands, Figure 7E). Even though IL-6 promoter does not have E-box sites, β-catenin can heterodimerize with MAX to bind to the TCFs/LCF1 element (Figure 7E). This suggests that MAX’s inductive effect may increase β-catenin’s expression and bind to the E-box element. Meanwhile, these results suggested that phospho S11 MAX may positively regulate the promoter activities by interacting with and enhancing the binding of β-catenin to the TCFs/LCF1 element, which positively regulated IL-6 transcription.

### MAX promotes HMGB1 and IL-6 through paracrine and autocrine mechanisms

**FIGURE 8:**
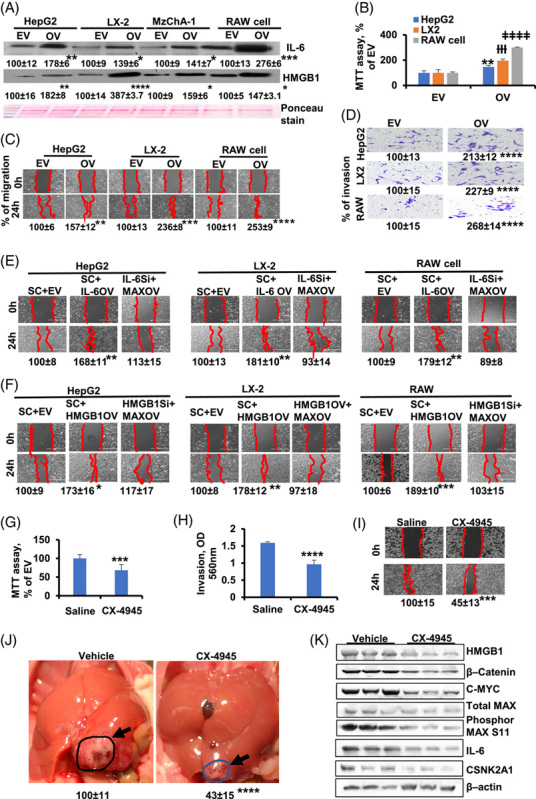
MAX promotes paracrine or autocrine of HMGB1 or IL-6, and CX-4945 treatment shows therapeutic efficacy in tumor growth in immunodeficient mice (A) The protein expression of HMGB1 and IL-6 in cell culture medium. Results are expressed as mean % of EV ± SEM, *p <0.05, **p <0.01, ***p <0.001, and ****p <0.0001 vs. EV. The coculture with overexpressed MAX in HepG2, LX2 and RAW cells were analyzed in MzChA-1 cells with MTT (B), migration (C), and invasion (D). Results for MTT, migration and invasion are shown as mean% of EV ± SEM; MTT: **p < 0.01, †††*p*<0.001, and ####*p* <0.0001 vs. EV; migration and invasion: **p  < 0.01, ***p <0.001, and ****p <0.0001 vs. EV. Data are shown in 3 different experiments (n=3). Media from IL-6 (E) and HMGB1 (F) knockdown or overexpression in HepG2, LX2 and Raw cell affects MzChA-1 migration, *p  < 0.05, **p  < 0.01, ***p < 0.001. The proliferation (G), invasion (H), and migration (I) of MzChA-1 in vitro after CX-4945 treatment. Results are mean % ± SEM of EV. ***p  < 0.001. ****p  < 0.0.01. (J) Tumor was developed by injecting immunodeficient mice with MzChA-1 cells per mouse through the left lobe of liver. Mice received vehicle or 100 mg/kg of CX-4945 through gavage twice daily from day 3 after injection MzChA-1 cells. The arrows point to representative tumor. **** *p* < 0.0001. (K) The protein expression of HMGB1, β-catenin, C-MYC, total MAX, phosphor-MAX S11, IL-6, and CSNK2A1 in MzChA-1 isolated from mouse tumor tissues with CX-4945 (left) and vehicle treatment (right).

To determine the role of overexpressed MAX on HMGB1 and IL-6 secretion from HepG2, LX2, MzChA-1, and RAW cells, we first analyzed the protein expression of HMGB1 and IL-6 in the cell culture medium. Of the 4 cell types examined, overexpression of MAX increased IL-6 release maximally from RAW cells, whereas HMGB1 release was the highest in LX2 cells (Figure 8A). Next, we investigated whether MAX overexpression in HepG2, LX2, and RAW cells promoted cell growth, migration, and invasion of MzChA-1 cells. We found that coculturing MzChA-1 with MAX overexpressing HepG2, LX2, or RAW cells markedly promoted cell growth (Figure 8B), migration (Figure 8C), and invasion (Figure 8D) compared with control (empty vector). Furthermore, media from HepG2, LX2, and RAW cell overexpressing IL-6 (Figure 8E) and HMGB1 (Figure 8F) measurably promoted MzChA-1 cell migration. In contrast, silencing of IL-6 (Figure 8E) or HMGB1 (Figure 8F) inhibited MzChA-1 cell migration.

### Effects of CX-4945 on CCA cell growth and in vivo tumorigenicity

To determine the effects of CX-4945 on MzChA-1 cell proliferation and invasion, we treated the cells at 10μM and examined the effect on cell growth. After 24h of treatment, CX-4945 reduced proliferation to 32% as compared with DMSO control group (Figure 8G). CX-4945 at 10 µM significantly reduced cell invasion to 39% as compared with DMSO control group (Figure 8H). Meanwhile, CX-4945 significantly inhibited cell migration (Figure 8I). To confirm their effects in vivo, the orthotopic tumorigenicity used injection of MzChA-1 cells into the left hepatic lobe as described.^21^ From day 3, these mice were treated with CX-4945 or a vehicle orally at 100 mg/kg twice daily for 27 days. The CX-4945-treated mice reduced tumor site to 67% (Figure 8J). Interestingly, CX-4945 treatment lowered HMGB1, β-catenin, C-MYC, phosphor-MAX, IL-6, and CSNK2A1 protein levels in tumor tissues in CX-4945 treatment as compared with a vehicle (Figure 8K).

## DISCUSSION

We originally developed a CCA mouse model by combining the chemical carcinogen DEN with partial duct ligation, which effectively simulates the multistep pathological evolution of human CCA from biliary proliferation to atypical hyperplasia, terminally developing into CCA. However, partial duct ligation is a relatively demanding technique for the operator and vulnerable to anesthetic and surgical risks. Long-term sustained furan exposure stimulates hepatocyte differentiation and induces irreversible bile duct lesions at high concentrations, or non-neoplastic bile duct lesions at lower concentrations. The latency period of these models varies (Supplemental Table S1, http://links.lww.com/HC9/A362).

The use of DEN in mice has become an attractive model for developing liver cancer. TAA enhances the carcinogenic effects of DEN-mediated tumorigenesis in the context of precancerous lesions. However, this TAA and DEN combination model has a low incidence of CCA and a high incidence of HCC.^25^ DEN administration results in the formation of multifocal biliary cystic lesions in mice and induces CCA in mice when combined with pentachlorophenol.^26^ The formation of a mixed CCA and HCC limits its applicability for studying iCCA. In this model, we used DEN oral gavage at a dose of 25 mg/kg (twice a week), which led to progression from biliary proliferation, atypical cystic hyperplasia, and cholangiomas to CCA. Moreover, this novel DEN model well mimics the pathophysiological features of CCA progression in humans.

CSNK2 is a highly conserved serine/threonine kinase involved in tumor biology by regulating cell proliferation, cell cycle progression, apoptosis, metabolism, and invasiveness.^8^ CSNK2A1 is highly expressed in cancer tissues such as gastrointestinal tract, head and neck, kidney, and prostate cancers.^9^ In this work, we found that CSNK2A1 protein levels were higher in hepatic cells from liver tissues with CCA. CSNK2A1 contributes to cancer progression by inducing phosphorylation of various molecules.^9,10,27^ We observed a positive correlation between CSNK2A1 and MAX phosphorylation. CSNK2A1 inhibitor CX-4945 also regulates the expression of more phosphor-MAX (S11) nuclear location. CX-4954 treatment can reduce the expression of MAX and MAX-interaction proteins and regulates also HMGB1 (−298/+1) and Il-6 (−491/+1) construct activity. Especially, CX-4945 inhibited MAX S11 phosphorylation and nuclear location. It is known that phosphorylation of MAX inhibits the DNA-binding activity of MAX homodimers but not C-MYC/MAX heterodimers.^24,28^ Since we found that CSNK2A1 phosphorylates MAX, we believe this may cause suppression of MAX-MAX homodimer and increase in MAX-C-MYC or MAX-β-catenin heterodimers and further upregulate HMGB1 and IL-6, favoring CCA progression.

MAX plays a vital role in the transcriptional regulation of MYC-targeted genes. We previously reported that the MYC-MAX heterodimer binds to E-box motifs and activates gene expression, whereas the heterodimer between MAX and MNT promotes transcriptional repression of cyclin D1.^5^ In this work, we discovered that MAX interacts with other proteins such as β-catenin, IL-6, and HMGB1 in CCA liver tissues, which in turn modulates transcription. C-MYC and MNT proteins in liver tissues rapidly degrade under normal conditions, with half-lives of 20 to 30 and 15 to 25 minutes, respectively.^29^ In contrast, MAX is a highly stable protein with a half-life of 24 hours in MzChA-1 cells and 48 hours in cholangiocytes isolated from liver tissues with CCA. Many studies demonstrated that protein phosphorylation affects its stability.^30^ Our experiment indicated that MAX phosphorylation may be associated with MAX stability since the phenomenon coexists in cholangiocytes that are isolated from CCA mice (Figures 2D, 5G). MAX promotes the growth, migration, invasion, and cell cycle progression of bile duct carcinoma cells. Even though MAX does not regulate CSNK2A1 expression, CSNK2A1 promotes MAX phosphorylation and nuclear localization.

There are 2 binding motifs (A and B boxes) in HMGB1, and the active cytokine domain of HMGB1 is localized to box B.^31^ HMGB1 plays a role in virus-induced biliary atresia pathogenesis and acts as a target for therapeutic intervention in some patients with biliary atresia and high HMGB1 expression.^32^ We found that higher HMGB1 expression persisted from biliary proliferation, atypical cystic hyperplasia, and cholangiomas to CCA. We also found that HMGB1 and MAX positively correlate with each other during CCA and interact with each other. Of the interacting proteins with MAX, HMGB1 is the primary MAX-interacting protein involved in CCA pathogenesis; its levels are increased in both the cell cytoplasm and the nucleus. Interestingly, we observed increased protein levels of HMGB1 in all cells isolated from CCA liver tissues of the DEN model including cholangiocytes, HSCs, KCs, and hepatocytes. Thus, HMGB1 upregulation in HSCs, KCs, hepatocytes, and cholangiocytes may be involved in regulating the balance between binding of MAX-C-MYC/MAX-β-catenin and MAX-MAX on the E-box site. Our findings also provide the rationale for inhibiting HMGB1 as a novel molecular targeted therapy of malignant CCA.

IL-6 is increased in the serum of patients with CCA and is a target molecule of CCA.^33^ Our data show that during DEN-treated CCA progression, IL-6 expression is upregulated in cholangiocytes, KCs, HSCs, and hepatocytes. Furthermore, MAX positively regulates IL-6 expression. Induction of MAX in HepG2, LX2, and RAW cells promoted migration of MzChA-1 cells, and silencing IL-6 or HMGB1 prevented MAX from exerting its positive effect on migration. These data strongly support the role of IL-6 and HMGB1 as downstream effectors of MAX. CX-4945 also lowers IL-6 expression and inhibits CCA cell growth, invasion, and migration in vitro and tumorigenesis in vivo. The results suggest that CX-4945 could be of therapeutic relevance in CCA through IL-6 downregulation.

Recent literature studies show that β-catenin signaling drives widespread gene repression and activation.^34^ We found that β-catenin expression in liver tissues during DEN treatment increased from week 1 onward. β-catenin protein expression was increased in cholangiocytes, KCs, HSCs, and hepatocytes. Phosphorylation of MAX inhibits the DNA-binding activity of MAX homodimers but not C-MYC/MAX heterodimers.^24,28^ On the basis of the interaction between β-catenin, C-MYC, and MAX and their co-occupancy on the HMGB1 promoter E-box, we believe that β-catenin-MAX may form heterodimers together with C-MYC-MAX heterodimers to regulate HMGB1 and IL-6 expression.

In conclusion, we have established a novel model of CCA progression from biliary proliferation to atypical cystic hyperplasia and to, eventually, cholangiocarcinogenesis. MAX acts as a master regulator through the upregulation of HMGB1, IL-6, C-MYC, and β-catenin in hepatic cells from liver tissues with CCA. C-MYC-MAX and β-catenin-MAX binding to E-box site or β-catenin-MAX bound to TCFs/LEF1 enhanced HMGB1 or IL-6 promoter activities. IL-6 and HMGB1 secreted by hepatocytes, HSCs, and KCs exert paracrine effects on cholangiocytes to promote cell growth, migration, and invasion and lead to the progression of cholangiocarcinogenesis. IL-6 and HMGB1 are downstream effectors of MAX. The combination of CSNK2A1 and MAX at S11 can regulate the occurrence of this series of processes. We propose that CX-4945 and MAX-interacting proteins provide perspectives on therapeutic strategies to attenuate progression from atypical cystic hyperplasia to cholangiocarcinogenesis.

### Statement of ethics

All animals were recruited in accordance with US law and institutional ethical guidelines. Samples were obtained from Cedars-Sinai Medical Center. The study protocol was approved by the US government and ethics committee.

## Supplementary Material

## References

[1] Arcognato S Sacchi D Fassan M Fabris L Cadamuro M Zanus G . Cholangiocarcinoma. Pathologica. 2021;113:158–69. 34294934 10.32074/1591-951X-252 PMC8299326

[2] Khan SA Davidson BR Goldin RD Heaton N Karani J Pereira SP . Guidelines for the diagnosis and treatment of cholangiocarcinoma: an update. Gut. 2012;61:1657–69. 22895392 10.1136/gutjnl-2011-301748

[3] Abdelrahim M Al-Rawi H Esmail A Xu J Umoru G Ibnshamsah F . Gemcitabine and cisplatin as neo-adjuvant for cholangiocarcinoma patients prior to liver transplantation: case-series. Curr Oncol. 2022;29:3585–94. 35621680 10.3390/curroncol29050290 PMC9139862

[4] Gutiérrez-Larrañaga M González-López E Roa-Bautista A Rodrigues PM Díaz-González Á Banales JM . Immune checkpoint inhibitors: the emerging cornerstone in cholangiocarcinoma therapy? Liver Cancer. 2021;10:545–60. 34950178 10.1159/000518104 PMC8647071

[5] Yang H Li TW Peng J Tang X Ko KS Xia M . A mouse model of cholestasis-associated cholangiocarcinoma and transcription factors involved in progression. Gastroenterology. 2011;141:378–88. 21440549 10.1053/j.gastro.2011.03.044 PMC3129489

[6] Cadamuro M Brivio S Stecca T Kaffe E Mariotti V Milani C . Animal models of cholangiocarcinoma: what they teach us about the human disease. Clin Res Hepatol Gastroenterol. 2018;42:403–15. 29753731 10.1016/j.clinre.2018.04.008

[7] Loeuillard E Fischbach SR Gores GJ Rizvi S . Animal models of cholangiocarcinoma. Biochim Biophys Acta Mol Basis Dis. 2019;1865:982–992. 29627364 10.1016/j.bbadis.2018.03.026 PMC6177316

[8] Meggio F Pinna LA . One-thousand-and-one substrates of protein kinase CK2? FASAB J. 2003;17:349–68. 10.1096/fj.02-0473rev 12631575

[9] Hussein UK Ahmed AG Song Y Kim KM Moon YJ Ahn AR . CK2α/CSNK2A1 induces resistance to doxorubicin through SIRT6-mediated activation of the DNA damage repair pathway. Cells. 2021;10:1770. 34359939 10.3390/cells10071770 PMC8303481

[10] Bae JS Park SH Jamiyandorj U Kim KM Noh SJ Kim JR . CK2α/CSNK2A1 phosphorylates SIRT6 and is involved in the progression of breast carcinoma and predicts shorter survival of diagnosed patients. Am J Pathol. 2016;186:3297–315. 27746184 10.1016/j.ajpath.2016.08.007

[11] Liu J Xiao Q Xiao J Niu C Li Y Zhang X . Wnt/β-catenin signalling: function, biological mechanisms, and therapeutic opportunities. Signal Transduct Target Ther. 2022;7:3. 34980884 10.1038/s41392-021-00762-6 PMC8724284

[12] Chen R Zhu S Fan XG Wang H Lotze MT Zeh HJ III . High mobility group protein B1 controls liver cancer initiation through yes-associated protein -dependent aerobic glycolysis. Hepatology. 2018;67:1823–41. 29149457 10.1002/hep.29663 PMC5906197

[13] Gaskell H Ge X Nieto N . High-mobility group box-1 and liver disease. Hepatol Commun. 2018;2:1005–20. 30202816 10.1002/hep4.1223 PMC6128227

[14] Lu M Qin X Zhou Y Li G Liu Z Yue H . LncRNA HOTAIR suppresses cell apoptosis, autophagy and induces cell proliferation in cholangiocarcinoma by modulating the miR-204-5p/HMGB1 axis. Biomed Pharmacother. 2020;130:110566. 32755793 10.1016/j.biopha.2020.110566

[15] Thongchot S Vidoni C Ferraresi A Loilome W Khuntikeo N Sangkhamanon S . Cancer-associated fibroblast-derived IL-6 determines unfavorable prognosis in cholangiocarcinoma by affecting autophagy-associated chemoresponse. Cancers (Basel). 2021;13:2134. 33925189 10.3390/cancers13092134 PMC8124468

[16] Augert A Mathsyaraja H Ibrahim AH Freie B Geuenich MJ Cheng PF . MAX functions as a tumor suppressor and rewires metabolism in small cell lung cancer. Cancer Cell. 2020;38:97–114. 32470392 10.1016/j.ccell.2020.04.016 PMC7363581

[17] Mathsyaraja H Freie B Cheng PF Babaeva E Catchpole JT Janssens D . Max deletion destabilizes MYC protein and abrogates Eµ-Myc lymphomagenesis. Genes Dev. 2019;33:1252–64. 31395740 10.1101/gad.325878.119 PMC6719623

[18] Comino-Méndez I Gracia-Aznárez FJ Schiavi F Landa I Leandro-García LJ Letón R . Exome sequencing identifies MAX mutations as a cause of hereditary pheochromocytoma. Nat Genet. 2011;43:663–7. 21685915 10.1038/ng.861

[19] Romero OA Torres-Diz M Pros E Savola S Gomez A Moran S . MAX inactivation in small cell lung cancer disrupts MYC-SWI/SNF programs and is synthetic lethal with BRG1. Cancer Discov. 2014;4:292–303. 24362264 10.1158/2159-8290.CD-13-0799

[20] Wang D Hashimoto H Zhang X Barwick BG Lonial S Boise LH . MAX is an epigenetic sensor of 5-carboxylcytosine and is altered in multiple myeloma. Nucleic Acids Res. 2017;45:2396–407. 27903915 10.1093/nar/gkw1184 PMC5389568

[21] Yang H Liu T Wang J Li TW Fan W Peng H . Deregulated methionine adenosyltransferase α1, c-Myc, and Maf proteins together promote cholangiocarcinoma growth in mice and humans(‡). Hepatology. 2016;64:439–55. 26969892 10.1002/hep.28541 PMC4956551

[22] Iwahasi S Rui F Morine Y Yamada S Saito YU Ikemoto T . Hepatic stellate cells contribute to the tumor malignancy of hepatocellular carcinoma through the IL-6 pathway. Anticancer Res. 2020;40:743–9. 32014916 10.21873/anticanres.14005

[23] Kewley RJ Whitelaw ML . Phosphorylation inhibits DNA-binding of alternatively spliced aryl hydrocarbon receptor nuclear translocator. Biochem Biophys Res Commun. 2005;338:660–7. 16129408 10.1016/j.bbrc.2005.08.073

[24] Bousset K Henriksson M Lüscher-Firzlaff JM Litchfield DW Lüscher B . Identification of casein kinase II phosphorylation sites in Max: effects on DNA-binding kinetics of Max homo- and Myc/Max heterodimers. Oncogene. 1993;8:3211–20. 8247525

[25] Li M Zhou X Wang W Ji B Shao Y Du Q . Selecting an appropriate experimental animal model for cholangiocarcinoma research. J Clin Transl Hepatol. 2022;10:700–10. 36062286 10.14218/JCTH.2021.00374 PMC9396327

[26] Mariotti V Strazzabosco M Fabris L Calvisi DF . Animal models of biliary injury and altered bile acid metabolism. Biochim Biophys Acta Mol Basis Dis. 2018;1864:1254–61. 28709963 10.1016/j.bbadis.2017.06.027 PMC5764833

[27] Zschoernig B Mahlknecht U . Carboxy-terminal phosphorylation of SIRT1 by protein kinase CK2. Biochem Biophys Res Commun. 2009;381:372–7. 19236849 10.1016/j.bbrc.2009.02.085

[28] Berberich SJ Cole MD . Casein kinase II inhibits the DNA-binding activity of Max homodimers but not Myc/Max heterodimers. Genes Dev. 1992;6:166–76. 1737614 10.1101/gad.6.2.166

[29] Conacci-Sorrell M McFerrin L Eisenman RN . An overview of MYC and its interactome. Cold Spring Harb Perspect Med. 2014;4:a014357. 24384812 10.1101/cshperspect.a014357 PMC3869278

[30] Potel CM Kurzawa N Becher I Typas A Mateus A Savitski MM . Impact of phosphorylation on thermal stability of proteins. Nat Methods. 2021;18:757–9. 34140700 10.1038/s41592-021-01177-5

[31] Jube S Rivera ZS Bianchi ME Powers A Wang E Pagano I . Cancer cell secretion of the DAMP protein HMGB1 supports progression in malignant mesothelioma. Cancer Res. 2012;72:3290–3301. 22552293 10.1158/0008-5472.CAN-11-3481 PMC3389268

[32] Mohanty SK Donnelly B Temple H Ortiz-Perez A Mowery S Lobeck I . High mobility group box 1 release by cholangiocytes governs biliary atresia pathogenesis and correlates with increases in afflicted infants. Hepatology. 2021;74:864–78. 33559243 10.1002/hep.31745 PMC8349381

[33] Mott JL Gores GJ . Targeting IL-6 in cholangiocarcinoma therapy. Am J Gastroenterol. 2007;102:2171–22. 17897336 10.1111/j.1572-0241.2007.01394.x

[34] Harmston N Lim JYS Arqués O Palmer HG Petretto E Virshup DM . Widespread repression of gene expression in cancer by a Wnt/β-Catenin/MAPK pathway. Cancer Res. 2021;81:464–75. 33203702 10.1158/0008-5472.CAN-20-2129

