# Supplementary figures and images for: CSNK2A1-mediated MAX phosphorylation upregulates HMGB1 and IL-6 expression in cholangiocarcinoma progression
Source: Hepatol Commun. 2023 Jun 22;7(7):e00144. doi: 10.1097/HC9.0000000000000144 (PMC10289747; doi:10.1097/HC9.0000000000000144)

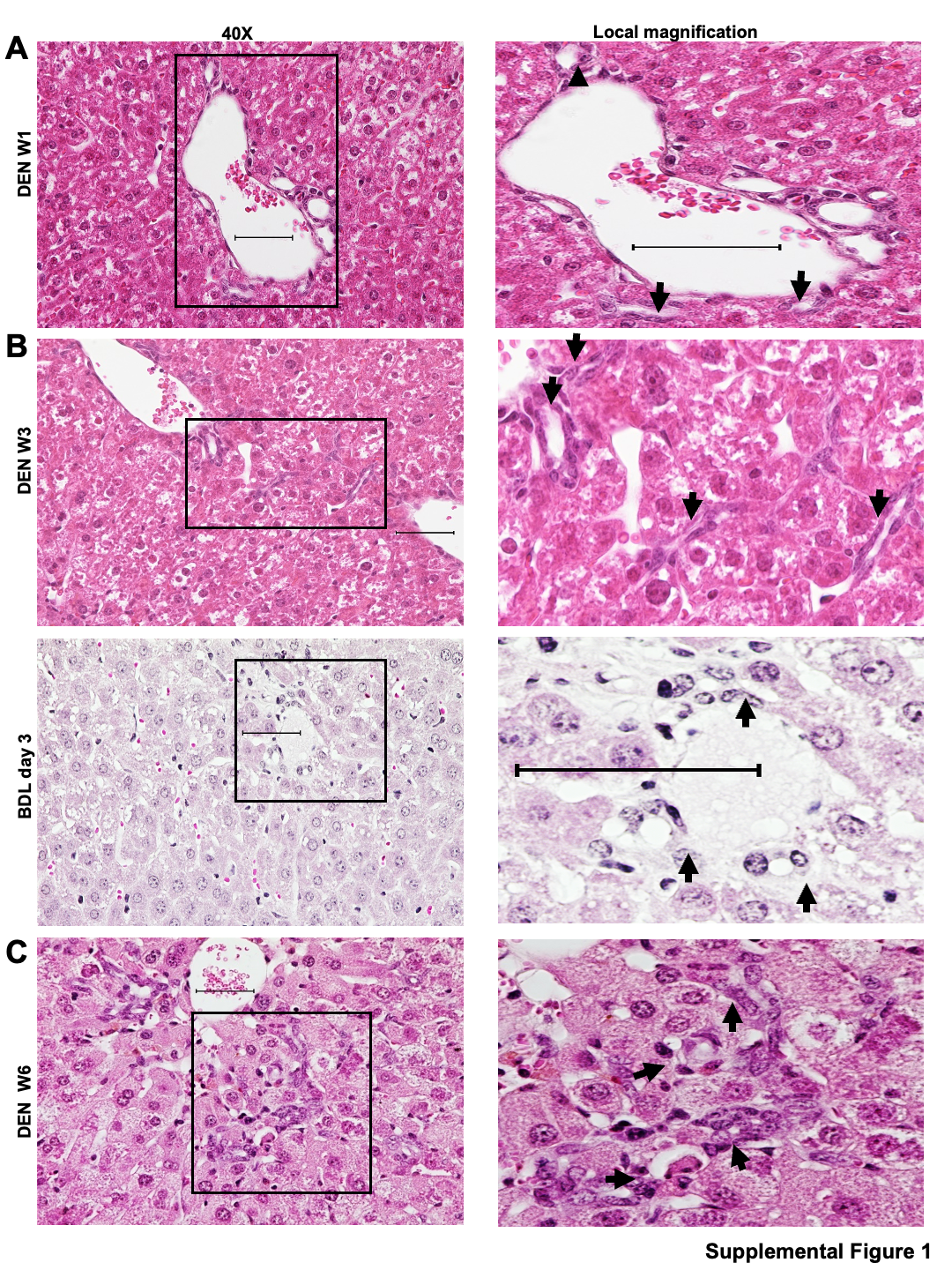

Supplement: Supplementary file 1 [file hc9-7-e00144-s001.tiff]

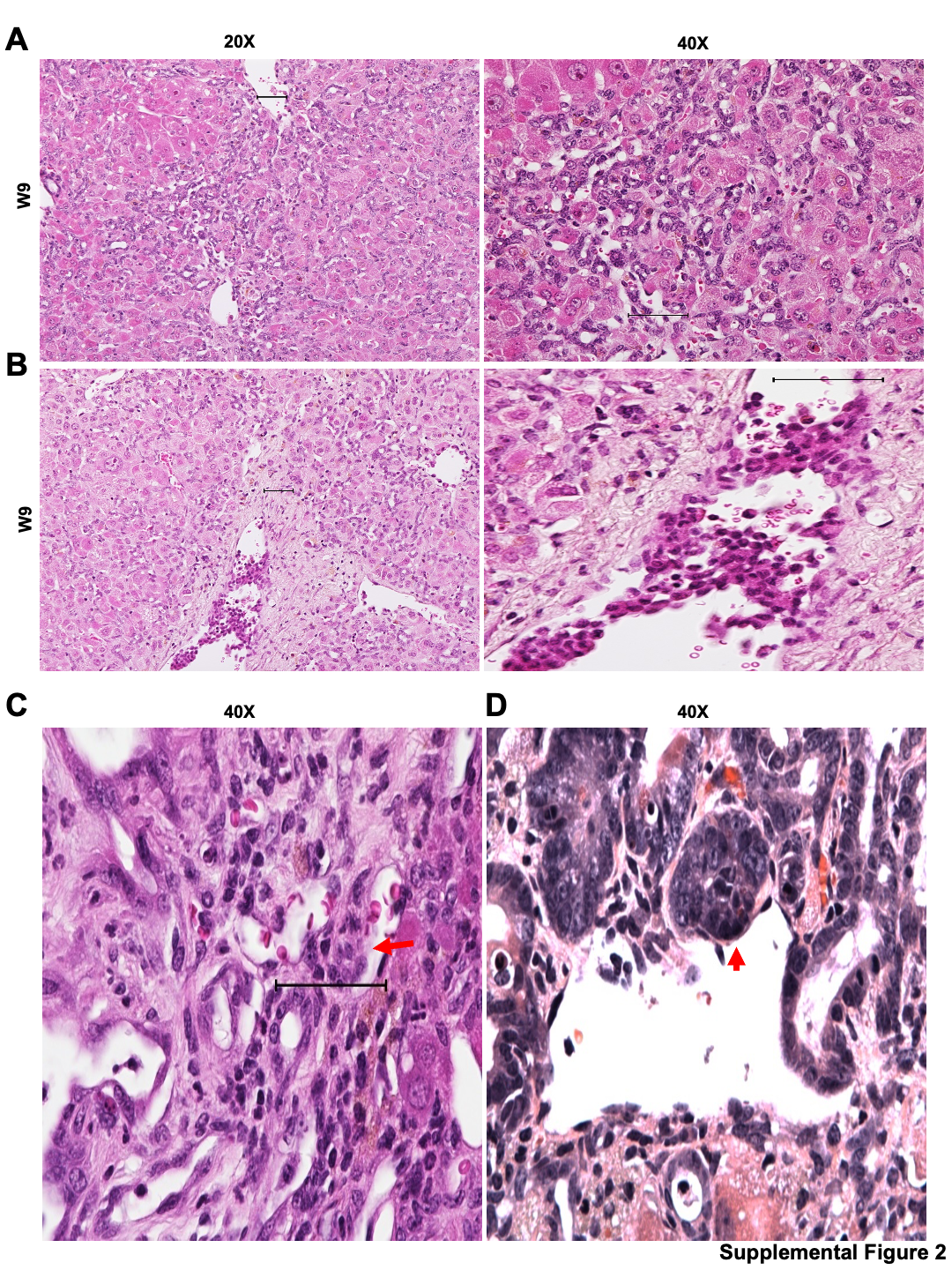

Supplement: Supplementary file 2 [file hc9-7-e00144-s002.tiff]

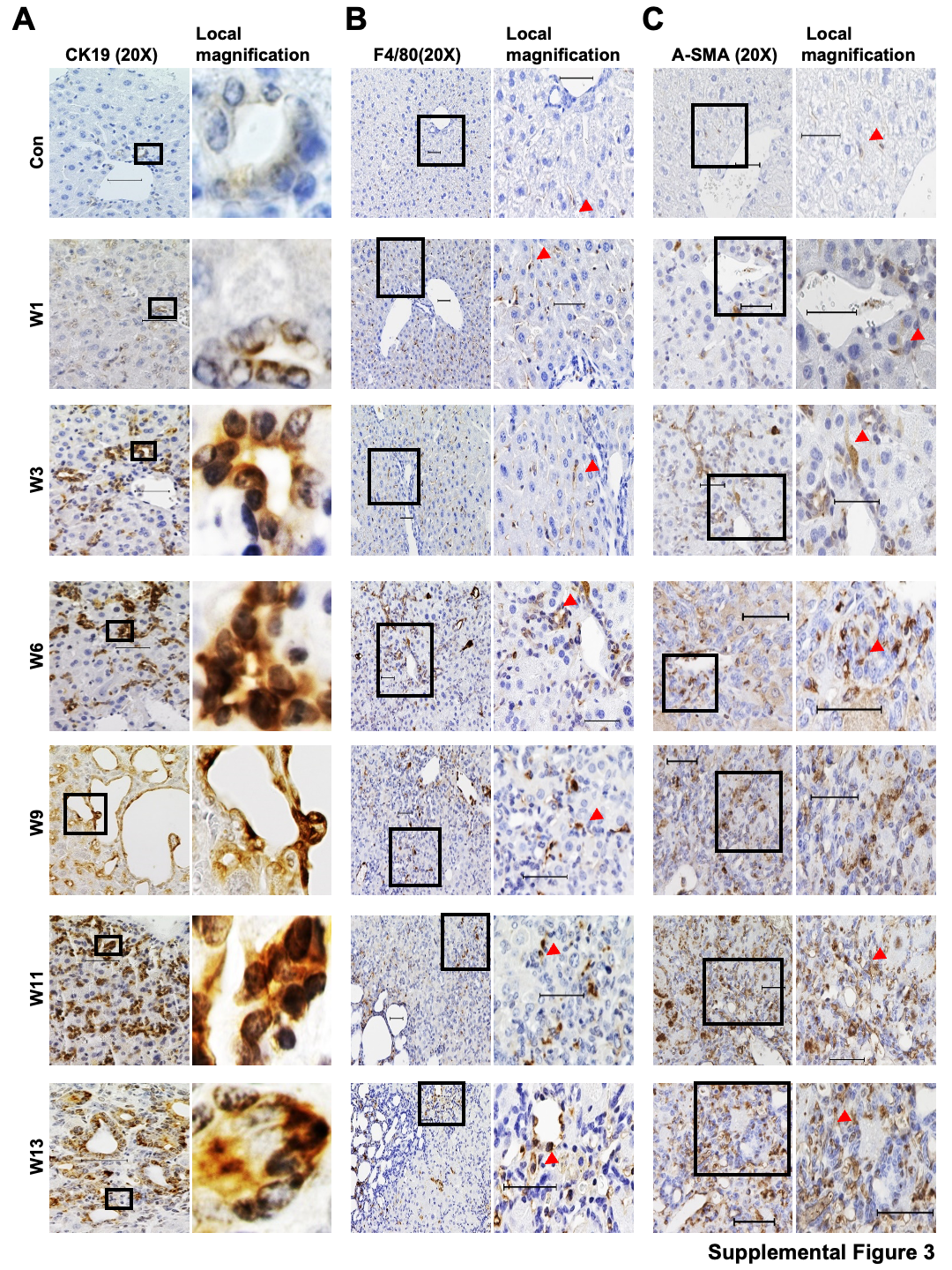

Supplement: Supplementary file 3 [file hc9-7-e00144-s003.tiff]

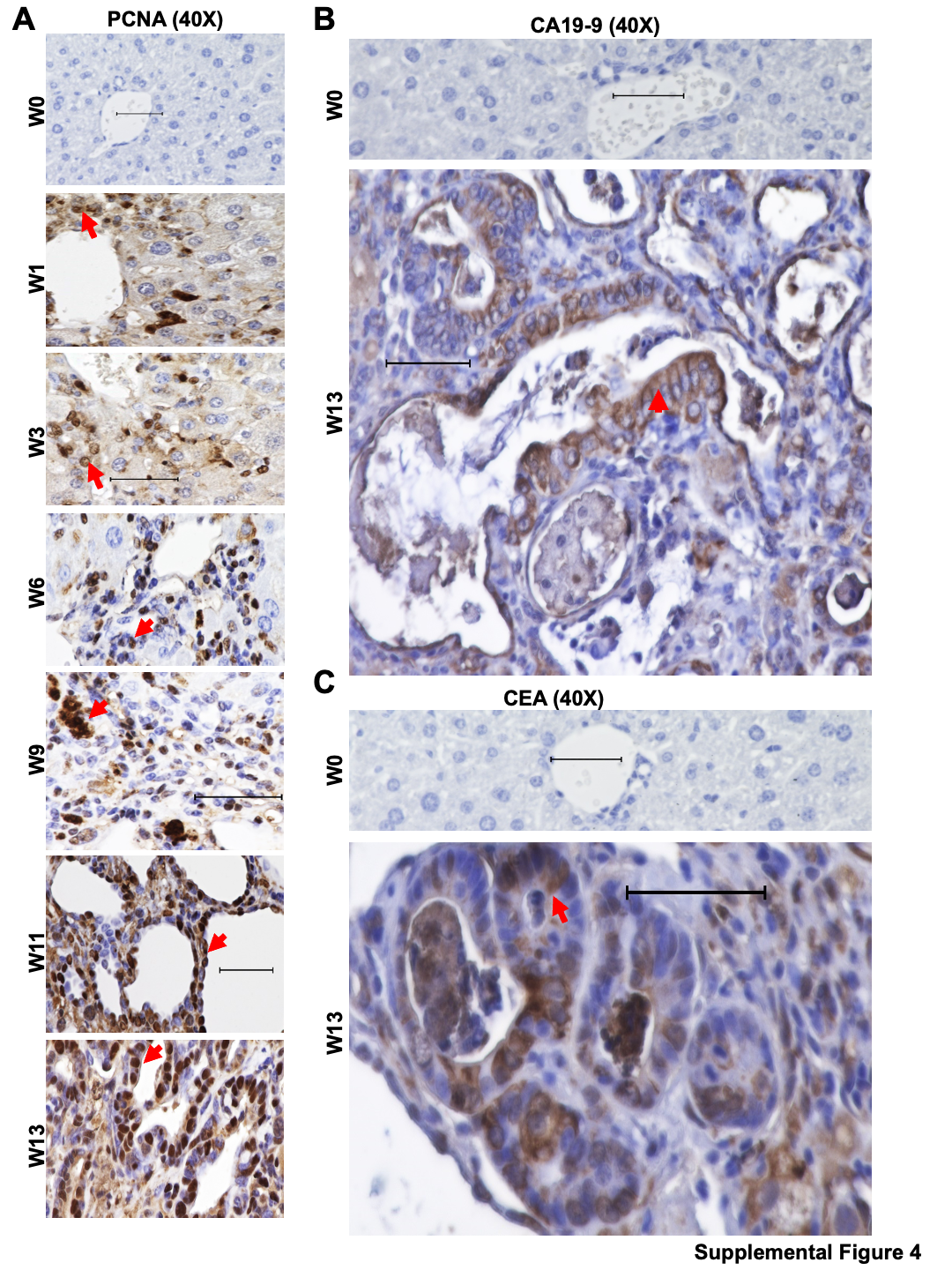

Supplement: Supplementary file 4 [file hc9-7-e00144-s004.tiff]

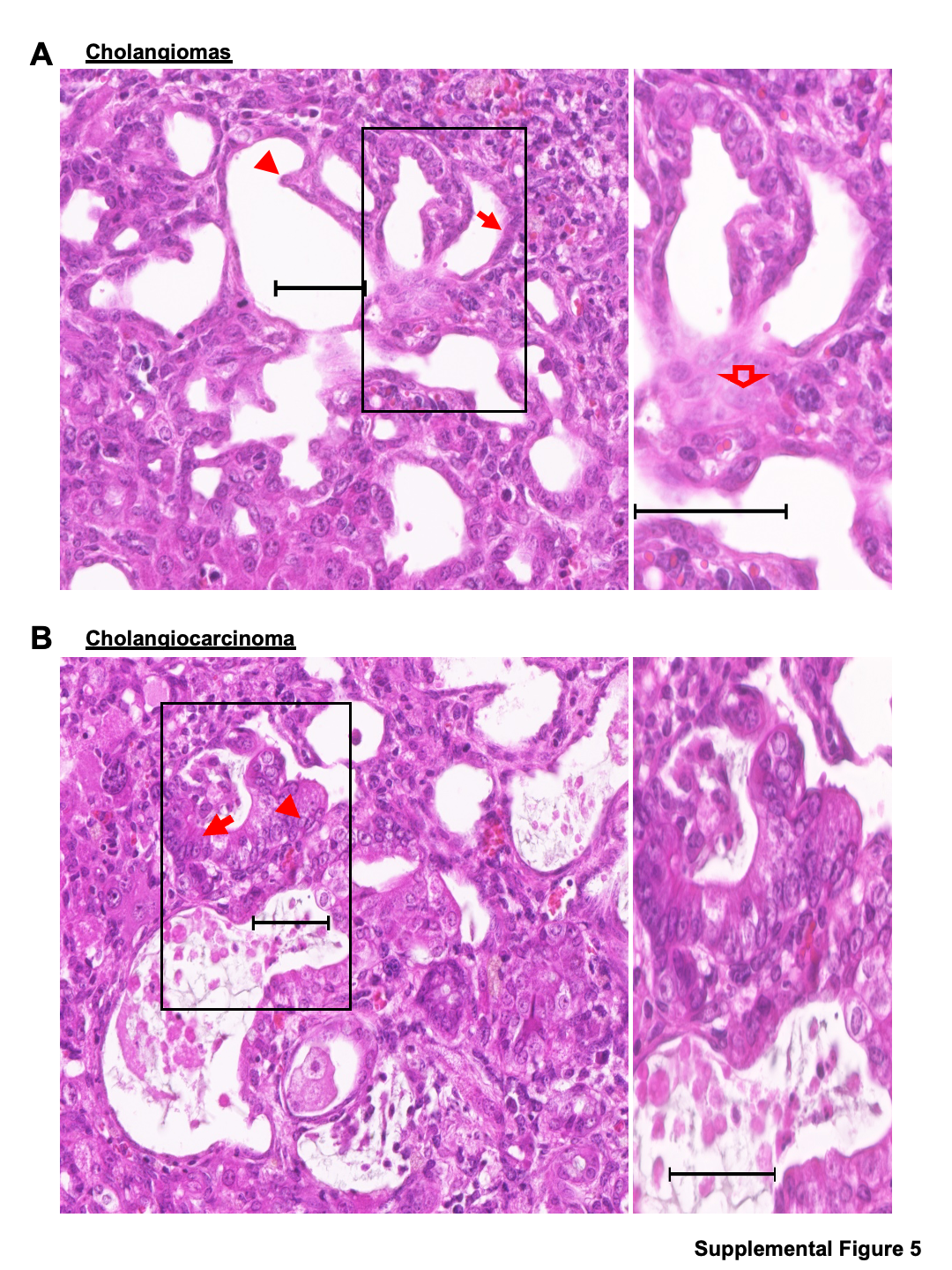

Supplement: Supplementary file 5 [file hc9-7-e00144-s005.tiff]

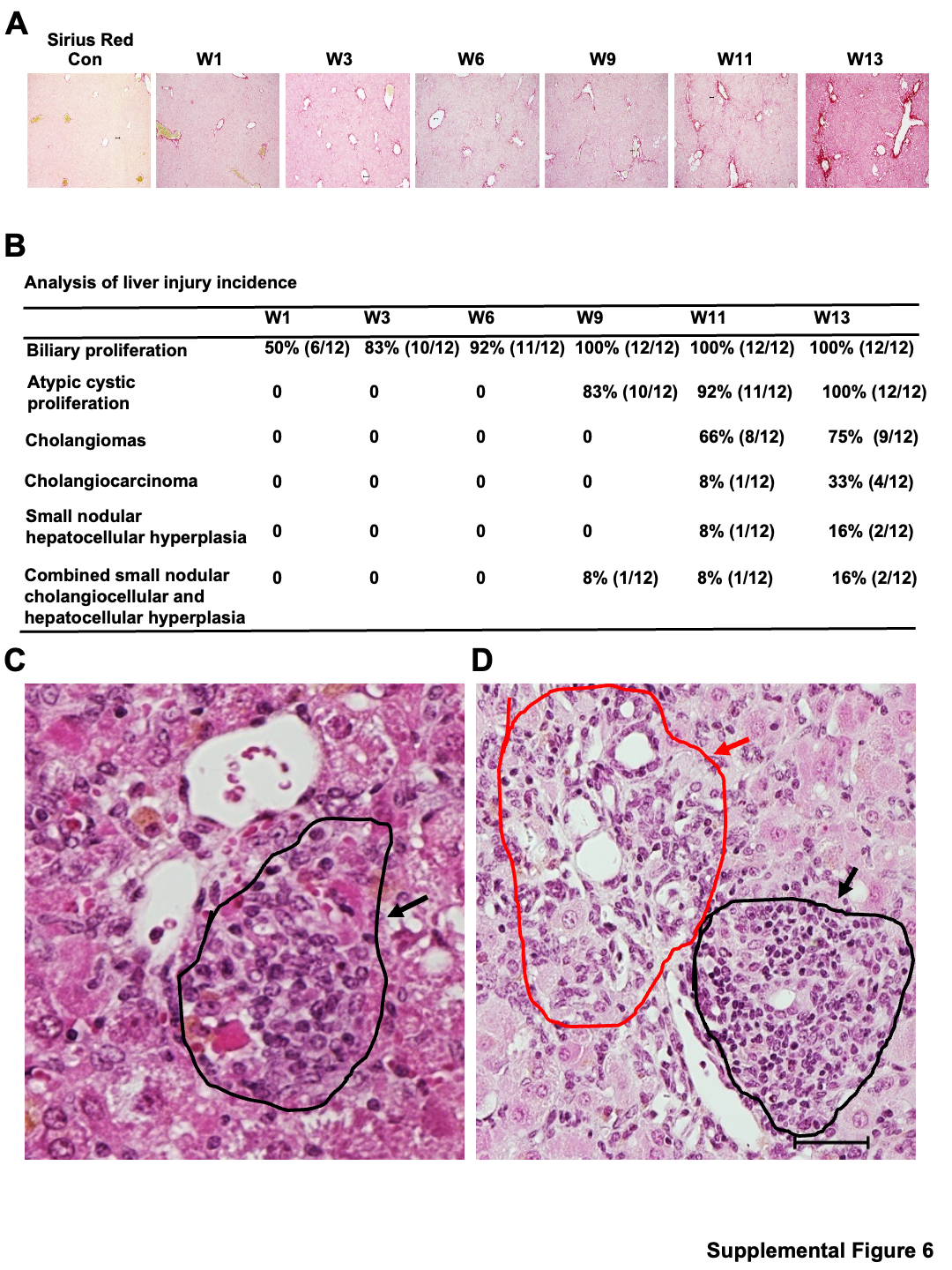

Supplement: Supplementary file 6 [file hc9-7-e00144-s006.tiff]

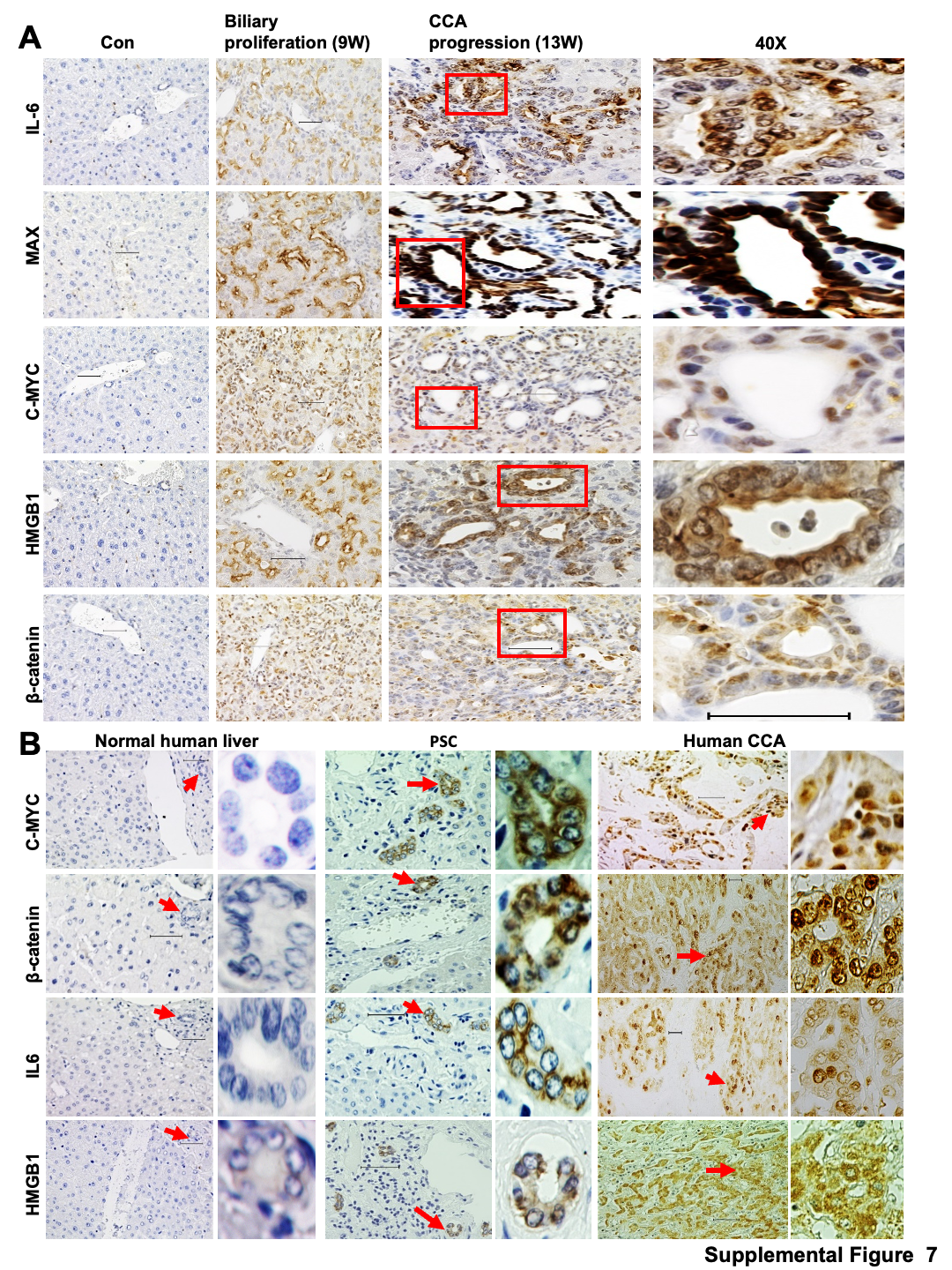

Supplement: Supplementary file 7 [file hc9-7-e00144-s007.tiff]

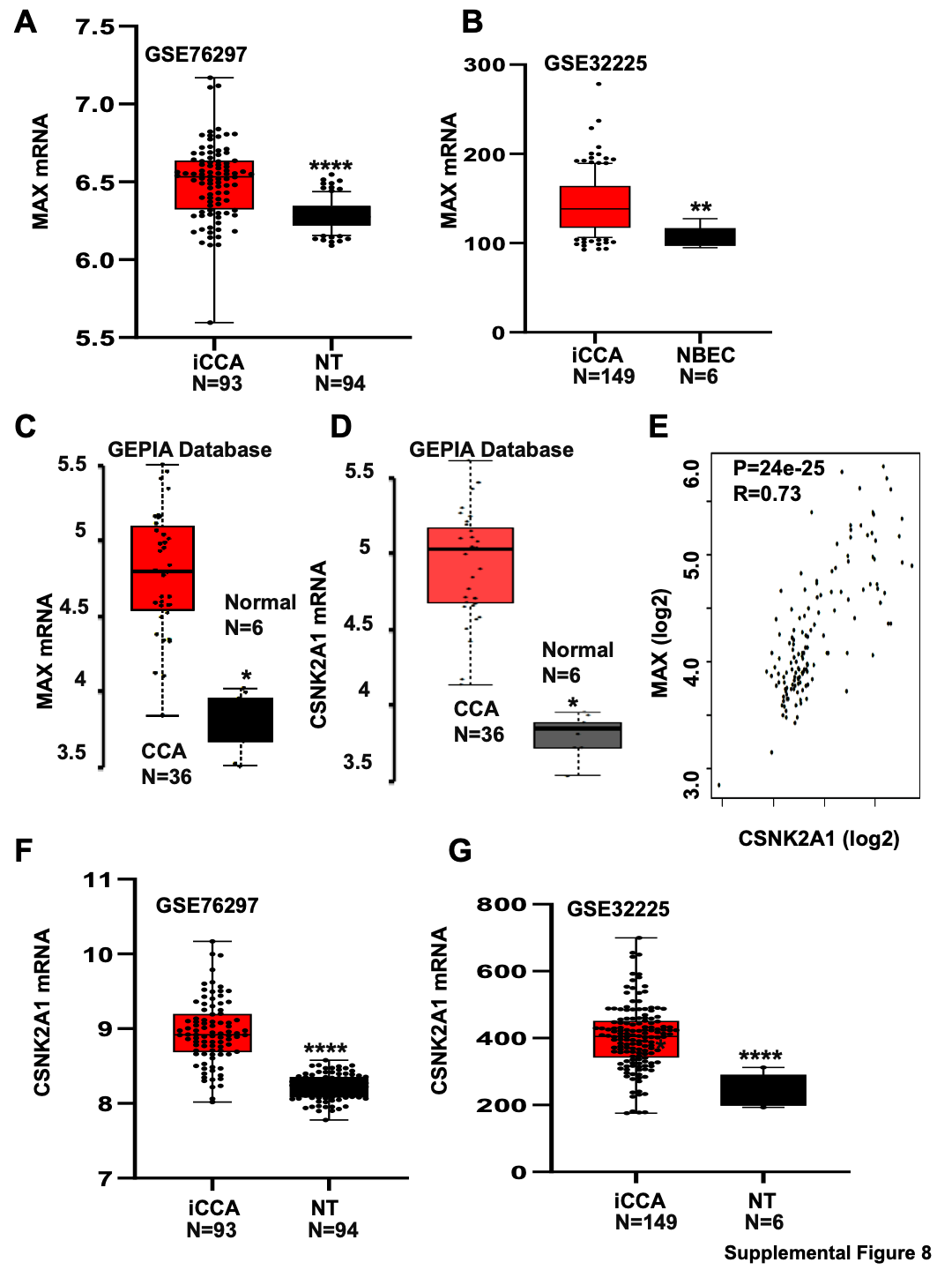

Supplement: Supplementary file 8 [file hc9-7-e00144-s008.tiff]

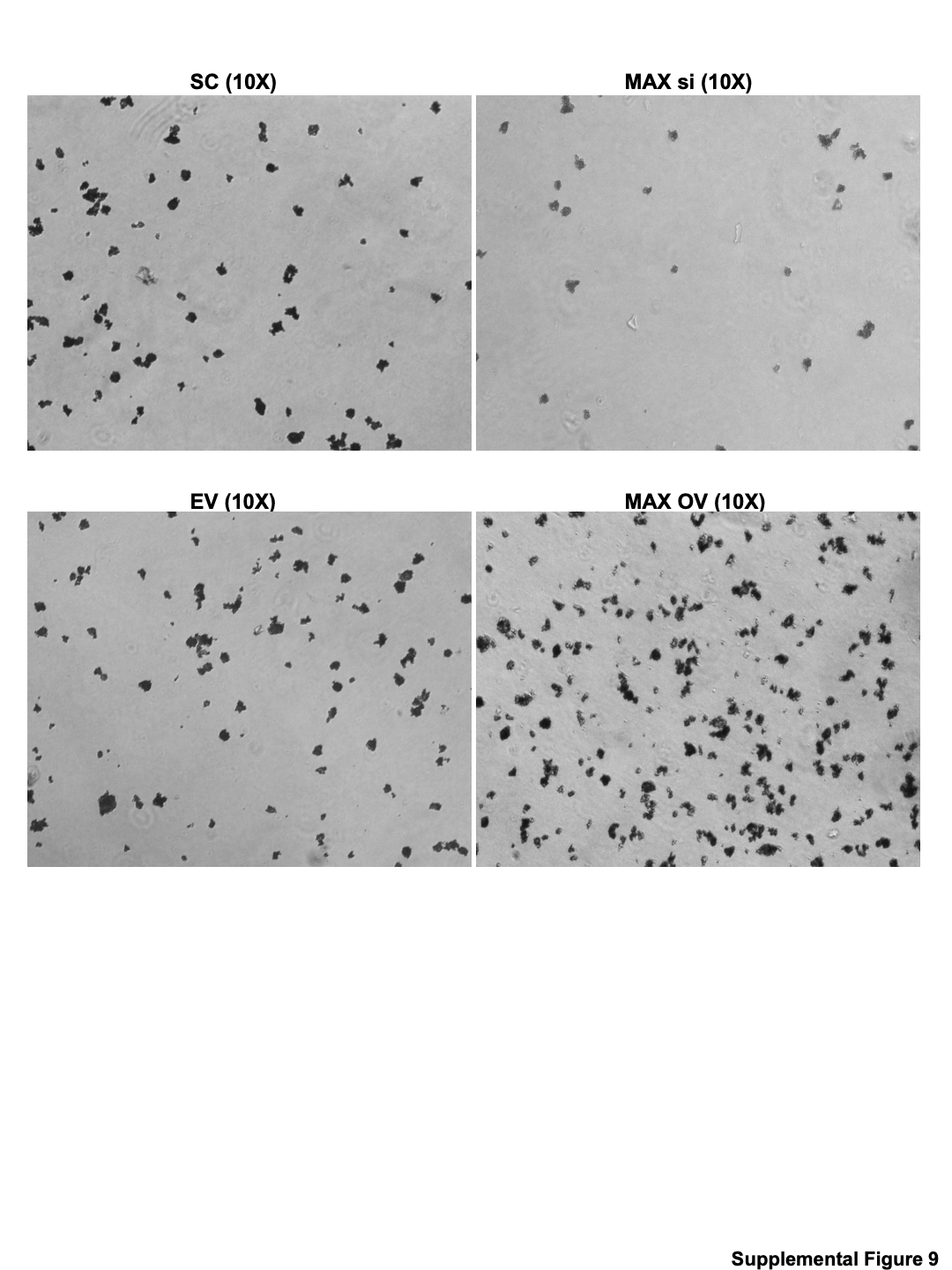

Supplement: Supplementary file 9 [file hc9-7-e00144-s009.tiff]

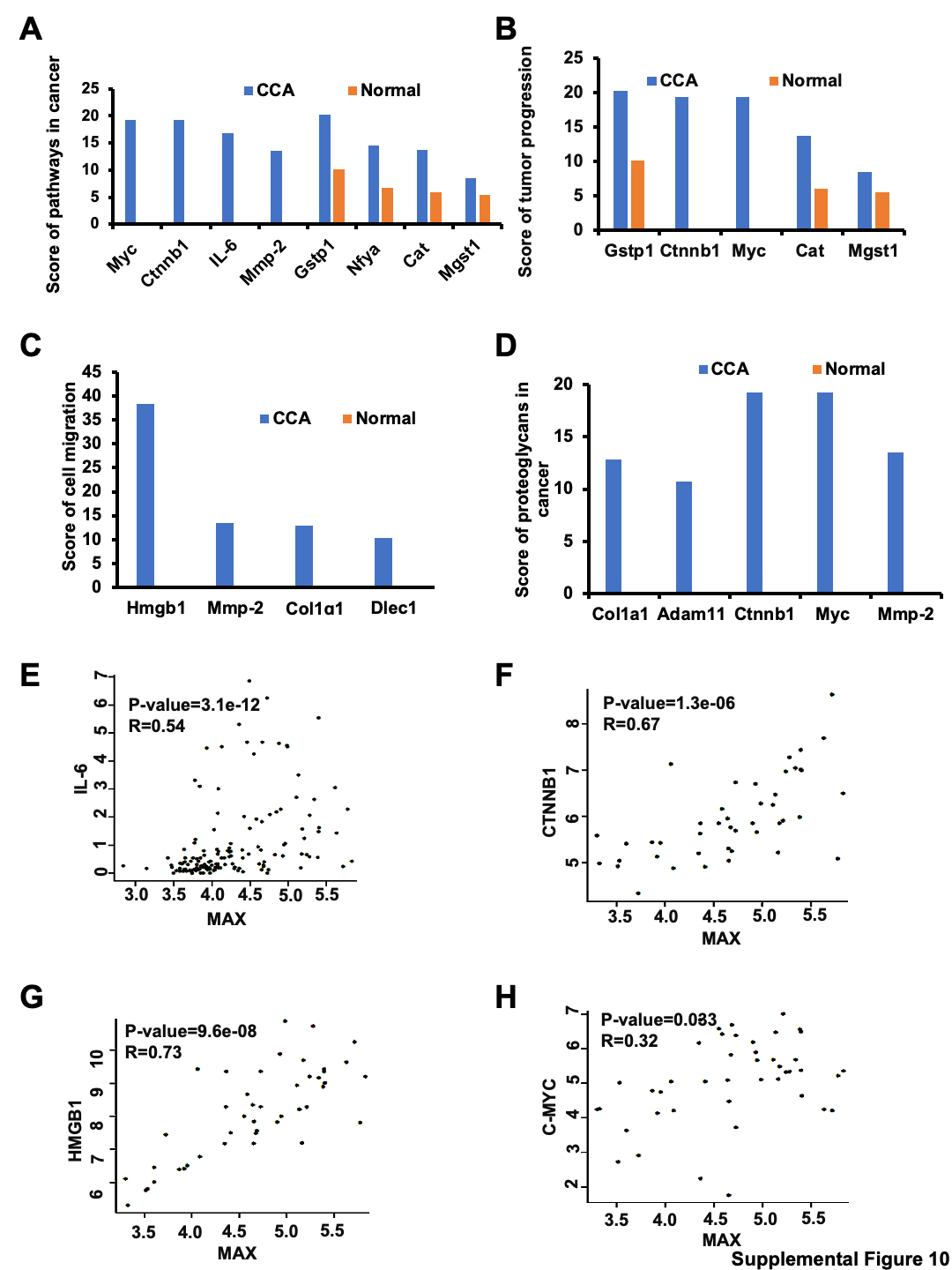

Supplement: Supplementary file 10 [file hc9-7-e00144-s010.tiff]

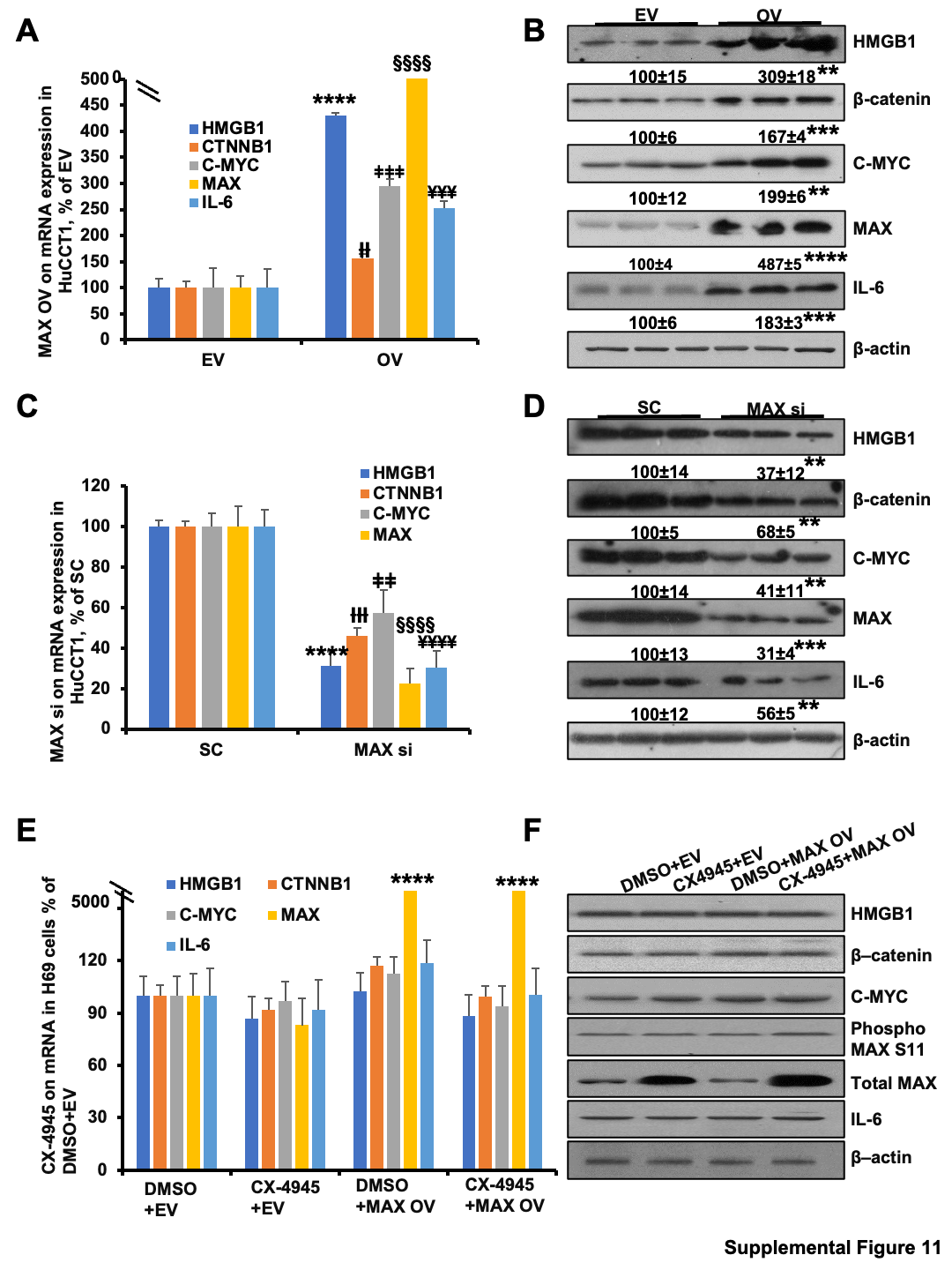

Supplement: Supplementary file 11 [file hc9-7-e00144-s011.tiff]

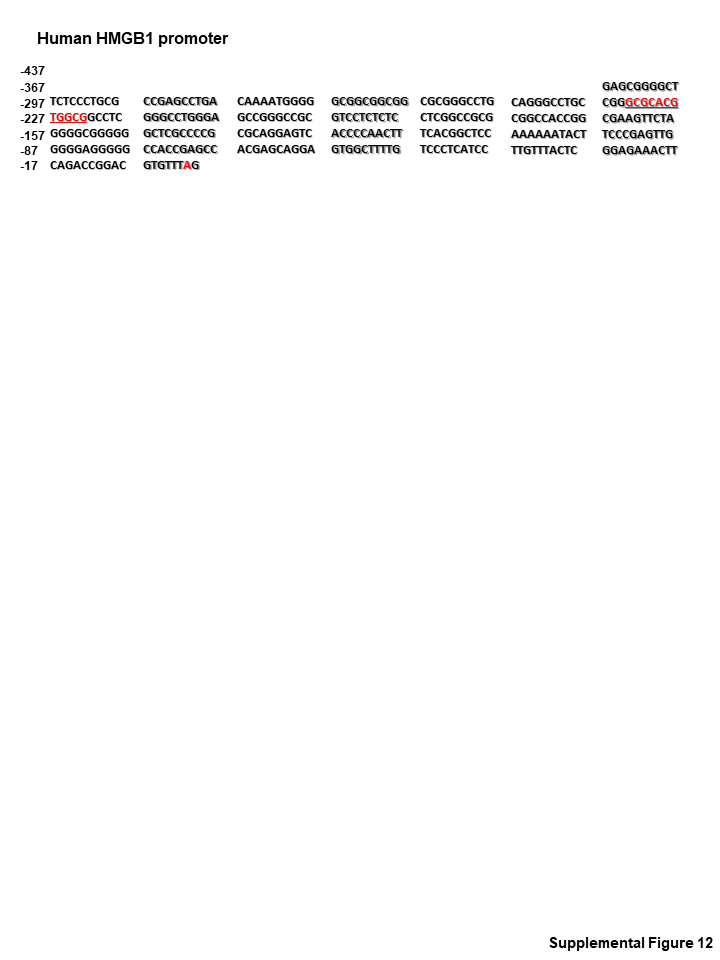

Supplement: Supplementary file 12 [file hc9-7-e00144-s012.tif]

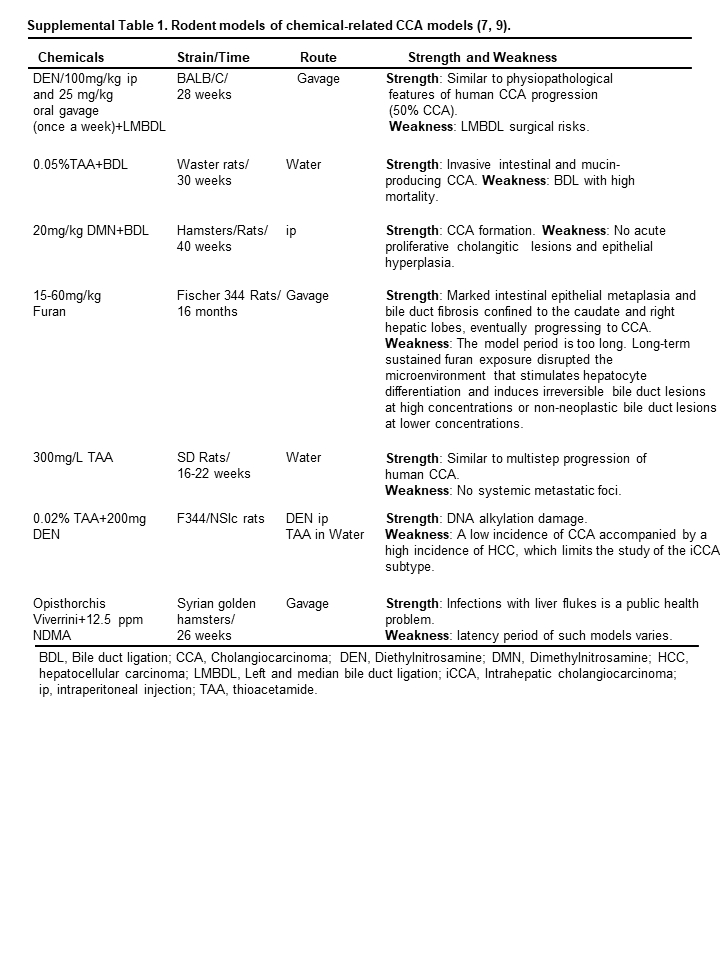

Supplement: Supplementary file 13 [file hc9-7-e00144-s013.tif]

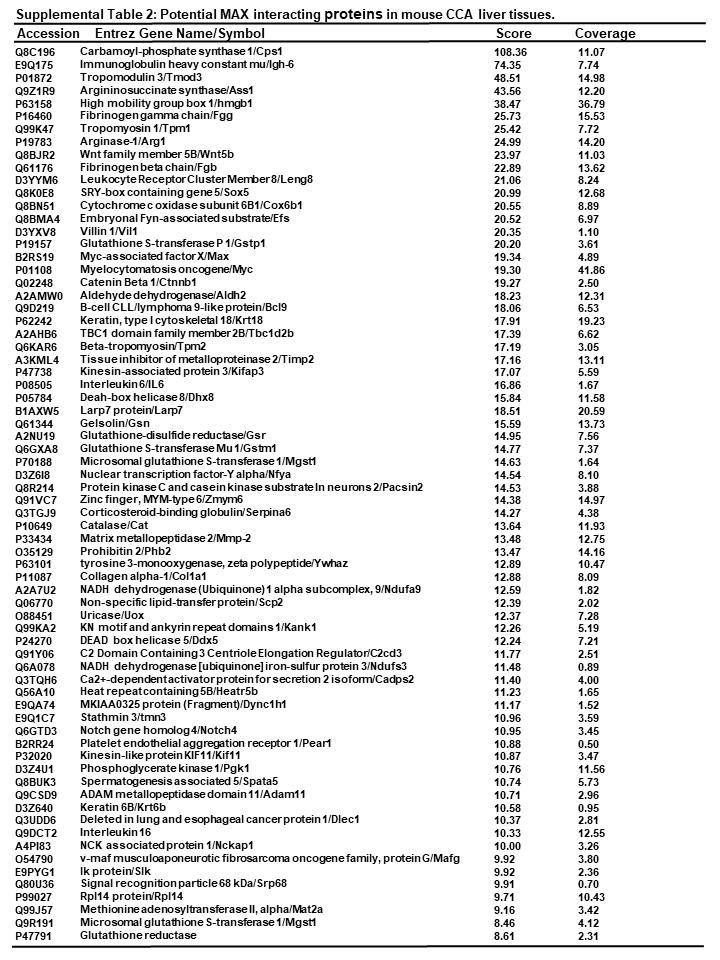

Supplement: Supplementary file 14 [file hc9-7-e00144-s014.tif]

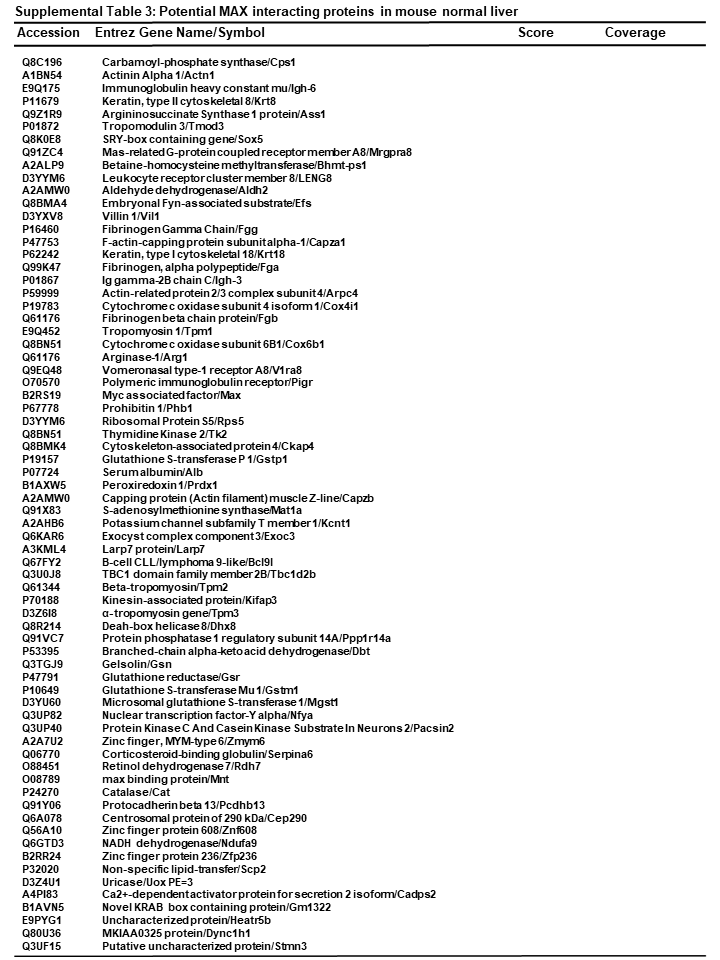

Supplement: Supplementary file 15 [file hc9-7-e00144-s015.tif]
